# Design and *in vitro* validation of Brome mosaic –virus-like particles for gene delivery and immunomodulation of melanoma

**DOI:** 10.1016/j.mtbio.2025.102693

**Published:** 2025-12-18

**Authors:** Khalil Elbadri, Manlio Fuscielo, Firas Hamdan, Ruoyu Cheng, Sara Feola, Honey Bokharaie, Carmine D'Amico, Giuseppina Molinaro, Alexandra Correia, Shiqi Wang, Michael Jeltsch, Vincenzo Cerullo, Hélder A. Santos

**Affiliations:** a Drug Research Program, Division of Pharmaceutical Chemistry and Technology, Faculty of Pharmacy, University of Helsinki, Helsinki, FI-00014, Finland; b Drug Research Program, Division of Pharmaceutical Biosciences, Faculty of Pharmacy, University of Helsinki, Viikinkaari 5E, Helsinki, FI-00014, Finland; c Orion Corporation, Turku, Finland; d Department of Bioproducts and Biosystems, Aalto University, 00076, Aalto, Finland; e Institute of Biotechnology, Helsinki Institute of Life Science, University of Helsinki, Helsinki, Finland; f Individualized Drug Therapy Research Program, Faculty of Medicine, University of Helsinki, Helsinki, FI-00014, Finland; g Wihuri Research Institute, Helsinki, FI-00014, Finland; h Helsinki One Health, University of Helsinki, Helsinki, FI-00014, Finland; i Department of Biomaterials and Biomedical Technology, The Personalized Medicine Research Institute (PRECISION), University Medical Center Groningen, University of Groningen, Ant. Deusinglaan 1, 9713 AV Groningen, the Netherlands

**Keywords:** siRNA delivery, Melanoma immunotherapy, PD-L1 silencing, Plant virus nanocarriers, Protein cages

## Abstract

RNA interference (RNAi) is a powerful tool for post-transcriptional gene silencing, yet its clinical translation remains limited by the lack of safe and efficient delivery systems. In this study, we evaluated the potential of plant virus-like particles (VLPs), derived from Brome Mosaic Virus (BMV), as a biodegradable and biocompatible nanocarrier for small interfering RNA (siRNA) delivery aimed at modulating immune checkpoints in melanoma. Recombinant BMV capsid proteins were expressed in *E. coli* and self-assembled *in vitro* into uniform VLPs encapsulating siRNA directed against the programmed death-ligand 1 (PD-L1). The VLPs displayed high structural stability, efficient siRNA encapsulation, and excellent biocompatibility. *In vitro*, A high cellular uptake was confirmed by confocal microscopy and flow cytometry. Besides, no cytotoxicity was observed and functional siRNA delivery was demonstrated *in vitro* by knockdown of eGFP in macrophages and PD-L1 in B16F10 melanoma and JAWS II dendritic cells, with significant knockdown efficiencies comparable to controls. Beyond molecular knockdown, PD-L1 silencing mediated by BMV-VLPs induced functional immunomodulation, increasing CD8⁺ T-cell–mediated cytotoxicity in melanoma and CMT64 tumor models and enhancing allogeneic T-cell proliferation in dendritic cell–based mixed leukocyte reactions. Overall, these findings indicate that plant-derived BMV-VLPs can safely mediate RNA-based checkpoint modulation and could complement antibody-based PD-1/PD-L1 blockade strategies. Owing to their intrinsic biocompatibility, scalability, and modular design, BMV-VLPs represent a sustainable and versatile alternative to synthetic lipid systems for RNA immunotherapy.

## Graphical abstract

In this study, we demonstrate the potential of *Brome mosaic* virus-derived virus like particles (BMV-VLPs), as a biocompatible siRNA carrier for silencing PD-L1, a key immune checkpoint, in melanoma cells. The BMV-VLPs exhibited efficient siRNA encapsulation, cellular uptake, and gene knockdown across tumor and immune cells, highlighting their potential as a versatile platform for RNA-based immunomodulation in cancer therapy.

**Figure:**
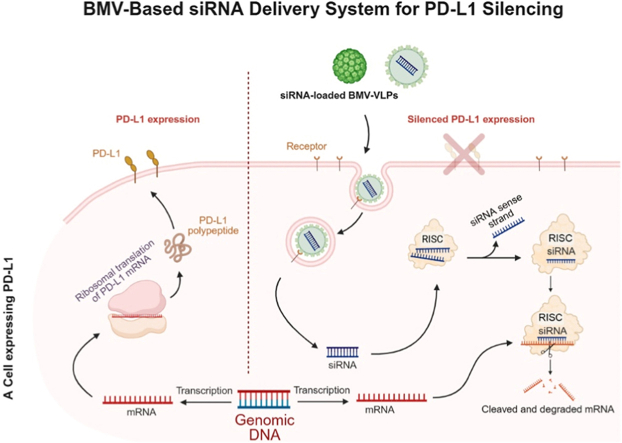

## 1 Introduction

Plant viruses have recently emerged as promising platforms for drug and gene delivery due to their structural uniformity, intrinsic biocompatibility, and non-pathogenic nature in mammals [[1], [2], [3]]. Unlike synthetic nanoparticles, which often require complex surface modifications to improve biocompatibility, stability and targeting, plant virus-based nanoparticles self-assemble into uniform nanoscale structures with inherent biocompatibility. In addition, unlike mammalian viral vectors, they are non-replicating in human cells and can be produced at scale in plants or bacteria, making them attractive for safe and cost-effective gene delivery in precision nanomedicine [[4], [5], [6]].

Several plant virus–derived nanoparticles, including Cowpea chlorotic mottle virus (CCMV) and Tobacco mosaic virus (TMV), have been extensively investigated as nanocarriers for imaging agents, vaccines, and nucleic acid delivery. These systems have demonstrated favorable biocompatibility and structural robustness; however, to our knowledge, most studies have focused on cargo delivery, imaging, or passive uptake, with limited exploration of functional immune modulation or immune checkpoint targeting. In particular, the application of plant virus–based VLPs for RNA interference–mediated modulation of tumor–immune interactions remains underdeveloped.

Among plant virus–based nanocarriers, virus-like particles (VLPs) derived from Brome Mosaic Virus (BMV) offer unique advantages for RNA delivery and functional immunomodulation due to their well-characterized icosahedral structure, reversible assembly, and intrinsic RNA encapsulation capability [7,8]. BMV forms VLPs of approximately 28 nm in diameter, composed of identical capsid protein (CP) subunits that can be expressed recombinantly and assembled *in vitro*, as well [7,9]. These VLPs have demonstrated the ability to encapsulate and protect a range of RNA molecules, including transfer RNA (tRNA) and small interfering RNA (siRNA), while maintaining structural stability [8,10]. BMV capsid assembly is reversible and responsive to environmental cues, such as pH and ionic strength. Hence, this pH- and ion-dependent behavior allows the capsid proteins to disassemble under high ionic or alkaline conditions and reassemble under physiological conditions. This feature enables controlled *in vitro* disassembly and reassembly of the viral capsids for RNA encapsulation [8].

Importantly, unlike many plant virus platforms that rely on plant-derived virions, BMV capsid proteins can be produced recombinantly and assembled *in vitro*, enabling modular design and precise control over cargo loading and surface properties [8]. Moreover, BMV is also highly adaptable; its capsid proteins can be chemically or genetically modified to display peptides, targeting ligands, or adjusting the surface charge, enabling precise tuning of cellular interactions and targeting specificity [10,11]. The capsid interior can accommodate structured RNA, double-stranded RNA, and even DNA nanostructures like DNA origami [12,13], reinforcing its potential as a modular protein cage for therapeutic nucleic acid delivery.

In addition to this structural and functional versatility, BMV-based nanoparticles exhibit favorable biocompatibility profiles. BMV-based nanoparticles displayed low immunogenicity in *in vitro* models [7], and have shown limited immune activation in rabbits [14]. However, recent *in vivo* studies suggest that repeated administration in mice may elicit an adaptive immune response [15]. While such immune activation may be undesirable in the context of systemic RNA delivery, controlled immunogenicity could be beneficial when BMV is used as an immunostimulatory platform, such as in cancer immunotherapy settings where adjuvanticity is desired. This highlights the need for immunomodulatory strategies to reduce immune activation, particularly in applications involving repeated dosing. This duality positions BMV as a delivery vehicle and also as a platform whose immune interactions can be strategically leveraged or minimized depending on therapeutic context.

Unlike other state-of-the-art delivery systems, such as lipid nanoparticles (LNPs), which often require pH-sensitive formulations, helper lipids, or fusogenic agents to facilitate cellular entry and endosomal escape [16], BMV VLPs exhibit intrinsic cell entry capabilities. Such intrinsic uptake ability, occurring without the need for helper lipids, fusogenic agents, or chemical transfection reagents, distinguishes plant virus–derived VLPs from synthetic lipid-based carriers. This naturally evolved capacity for cellular internalization, together with their uniform nanoscale structure and excellent biocompatibility, positions BMV-VLPs as promising candidates for repeatable and well-tolerated gene delivery, particularly in the context of cancer immunotherapy and checkpoint modulation.

Although the precise mechanism remains under investigation, the current evidence suggests that several plant and bacteriophage VLPs enter mammalian cells primarily through endocytic pathways, including clathrin-mediated endocytosis [17] and, in some cases**,** caveolae-dependent endocytosis [18]. Both pathways can facilitate the intracellular trafficking and functional delivery of encapsulated RNA cargo [11,19].

Altogether, these features position BMV as a compelling alternative to synthetic platforms such as LNPs, particularly in applications requiring precise, repeatable, and well-tolerated gene delivery, such as cancer immunotherapy for melanoma.

Melanoma, a highly immunogenic form of skin cancer, often exploits immune checkpoint pathways to evade T cell-mediated destruction [21]. The global burden of melanoma continues to rise, with increasing incidence and mortality rates, particularly in regions with high ultraviolet exposure [22]. The disease imposes a significant economic burden due to the high costs of long-term immunotherapies and the clinical management of metastatic cases [23,24]. During tumor progression, the programmed cell death protein 1 (PD-1), an immune checkpoint protein, plays a key role in melanoma immune evasion by suppressing T cell activity through binding to its ligand, programmed death ligand 1 (PD-L1), that becomes overexpressed on tumor and immune cells. While anti-PD-1 and anti-PD-L1 monoclonal antibodies have revolutionized melanoma therapy [25,26], their use is limited by cost, immune-related side effects, and inconsistent patient responses [21]. As an alternative, siRNA-mediated silencing of PD-L1 offers a precise and tunable approach to modulate immune checkpoints at the gene level [27,28]. Yet, clinical translation of siRNA is constrained by instability and poor cellular uptake [29]. To overcome these limitations, VLPs derived from plant virus, such as those derived from BMV, offer a promising RNA delivery platform due to their biocompatibility, cargo protection, and cell entry efficiency, making them ideal candidates for delivering therapeutic siRNA targeting PD-L1.

Although BMV-based VLPs have previously been used for siRNA delivery, their application specifically to immune checkpoint modulation and the functional validation of T-cell responses has not, to our knowledge, been reported. In this study, we evaluated recombinant Brome Mosaic Virus–like particles (BMV-VLPs) as biodegradable nanocarriers for delivering functional siRNA to modulate PD-L1–dependent immune suppression in melanoma and immune cell models. Recombinant BMV capsid proteins (rCPs) were expressed in *E. coli*, purified, and validated by ELISA for correct antigenic folding, then self-assembled *in vitro* into uniform siRNA-loaded VLPs. Using both model green fluorescent protein (eGFP) and therapeutic (PD-L1) siRNAs, we assessed encapsulation efficiency, delivery performance, and gene-silencing activity across melanoma cells, macrophage-like cells, and dendritic cells. Beyond molecular knockdown, we further examined whether PD-L1 silencing restored antigen-specific T-cell cytotoxicity and enhanced allogeneic T-cell activation. Collectively, this work establishes BMV-VLPs as a plant virus–derived RNA delivery platform capable of both targeted gene silencing and functional immunomodulation, providing a promising foundation for RNA-based cancer therapeutics.

## 2 Results and discussion

### 2.1 Production, purification and characterization of wild-type BMV nanoparticles

To establish a comparative baseline for engineered VLPs, *wt*BMV was propagated in barley plants via mechanical inoculation using homogenates of infected leaves. By 12 days post-inoculation (dpi), characteristic chlorotic and mosaic patterns were observed on infected leaves (Fig. S1A–C), consistent with BMV infection pattern described elsewhere [30,31]. These visual markers confirmed successful viral replication and systemic infection in the host.

Viral particles were extracted from symptomatic leaves, and crude plant sap was processed via differential centrifugation to remove debris. Further purification was achieved through a two-step ultracentrifugation process: a sucrose cushion followed by a sucrose gradient, which enabled separation of intact virions from host proteins and other impurities (Fig. S1D–F).

The integrity and purity of the purified *wt*BMV particles were validated by Sodium Dodecyl Sulfate-Polyacrylamide Gel Electrophoresis (SDS-PAGE), which showed a prominent band at ∼20 kDa corresponding to the BMV CP (Figs. S1G and S3A). This was consistent with the expected molecular weight of the viral coat protein. Additionally, virus-specific ELISA confirmed strong immunoreactivity in extracts collected at the end of the first- and second-weeks post-inoculation, as well as in a +ve control consisting of lyophilized naturally infected tissue obtained from Agdia (USA) (Fig. S1H), indicating high viral titers and confirming the presence of BMV CP in the purified samples. These results indicate successful expression and recovery of viral components. However, the structural integrity of the particles was further confirmed by transmission electron microscopy (TEM) and dynamic light scattering (DLS) for size consistency, as confirmed in the following sections. These results established the efficient propagation and purification of *wt*BMV, providing a reference standard for evaluating the structure, size, and cargo-loading efficiency of recombinant BMV-derived VLPs in the subsequent experiments.

### 2.2 Recombinant BMV capsid protein expression and VLP assembly

#### 2.2.1 Biochemical characterization; ELISA and SDS-PAGE

Recombinant BMV-rCP was successfully expressed in *Escherichia coli* (*E. coli*) using pMCSG48 vector system (Fig. S2A). and purified for downstream assembly. To evaluate antigenic integrity, ELISA was performed using BMV-specific antibodies. Strong reactivity was observed for both *wt*BMV and the assembled recombinant VLPs, whereas uninduced bacterial lysates had a negligible signal (Fig. S2B). These results confirm that the rCP retains conformational epitopes characteristic of the native virion. SDS-PAGE analysis of the final purified product revealed a dominant band at ∼20 kDa, consistent with the expected molecular weight of tag-free BMV coat protein and matching the size of native capsid subunits reported in prior studies [7,8] (Figs. S2C and S3B).

#### 2.2.2 Virus-like particle construction

As mentioned above, the assembly/disassembly of the BMV capsid is regulated by environmental factors, such as pH, ionic strength, and the presence of divalent cations (*e.g.,* Mg^2+^) [8] (Fig. S2D–F). These conditions critically influence the stability and integrity of the capsid, allowing for precise control over its assembly and disassembly processes. Specifically, disassembly is favored under alkaline and low ionic strength conditions [20,32], where increased electrostatic repulsion disrupts protein–protein and protein–RNA interactions. Alternatively, capsid destabilization may also occur at neutral pH when accompanied by high ionic strength, which competes with RNA for electrostatic interactions, leading to RNA release or particle fragmentation [9]. In contrast, capsid assembly is favored under moderate ionic strength and near-neutral pH, where divalent cations (*e.g.,* Mg^2+^) neutralize electrostatic repulsion and promote stable protein–protein interactions [33]. Likewise, BMV-VLPs also were found to maintain structural integrity under mildly acidic environments (pH ∼4.5), further emphasizing their environmental responsiveness [32].

To aid visualization, a structural model of the BMV-VLP was generated in UCSF ChimeraX using published crystallographic coordinates and capsid architecture data [8,9,34] (Fig. S2D–H). The model was used solely for conceptual illustration and was not derived from experimental structural analysis. It highlights the icosahedral T = 3 lattice composed of quasi-equivalent A, B, and C conformers stabilized by Mg^2+^-mediated interactions. The resulting cage contains surface pores and internal cavities that can accommodate RNA cargos, illustrating its potential as a modular nanocarrier for nucleic acid delivery or displaying functional moieties [9].

In the following section, we established the *in vitro* assembly of recombinant BMV-VLPs around siRNA cargos, together with evaluating their biophysical characterization and encapsulation efficiency, to assess their suitability as RNA delivery platforms for melanoma therapy.

#### 2.2.3 Biophysical characterization of BMV VLPs; DLS and TEM

BMV's multipartite genome consists of four single-stranded positive-sense RNA molecules of various sizes—RNA1 (∼3.2 kb), RNA2 (∼2.8 kb), RNA3 (∼2.1 kb), and RNA4 (∼0.8 kb), which are selectively encapsulated into separate particles [9,35]. To investigate the RNA-packaging behavior of recombinant BMV capsid proteins, we assembled VLPs encapsulating various RNAs; yeast tRNA and synthetic siRNAs targeting eGFP and PD-L1, as model cargos. The self-assembly of rCPs into VLP was initiated *in vitro* under near-physiological conditions. These RNAs were selected for their relevance in RNA delivery applications and their structural diversity [36,37]. The assembled particles were analyzed using DLS and TEM to assess size distribution, uniformity, and morphology in comparison to *wt*BMV.

**Fig. 1:**
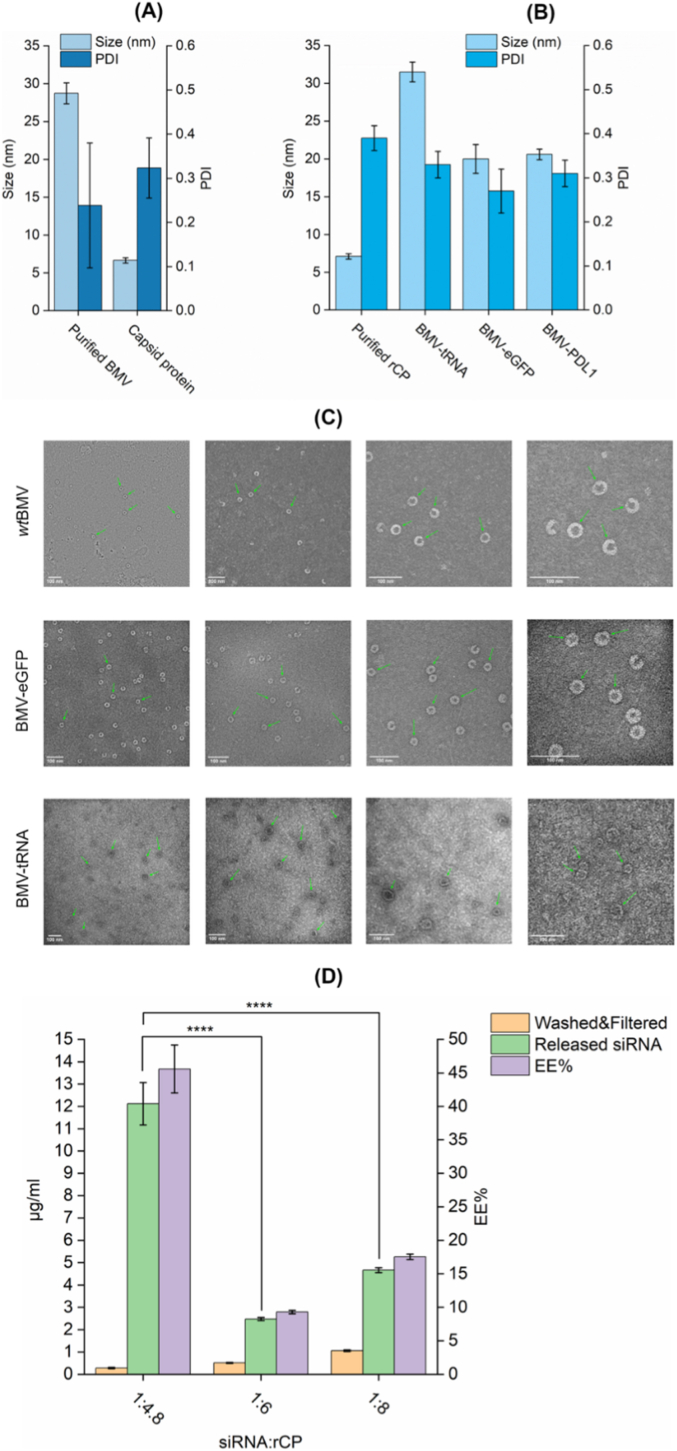
Encapsulation of different RNAs in BMV-VLPs and their biophysical characterization. (A,B) DLS analysis of purified *wt*BMV, dissociated CP, rCP, and RNA-loaded VLPs. *wt*BMV and VLPs assembled with yeast tRNA showed a comparable size of ∼28 nm and ∼32 nm size, respectively, while those loaded with siRNA (eGFP or PD-L1) exhibited smaller diameters (∼20 nm), reflecting cargo-dependent structural differences. (C) Negative-stain TEM images showing well-defined, spherical morphology of *wt*BMV and recombinant VLPs loaded with either tRNA or siRNAs. Green arrows indicate representative intact particles. The apparent size differences among samples were less pronounced in TEM micrographs, partly because different magnifications were applied to enhance particle visibility and due to inherent methodological differences between TEM (dry-state physical diameter) and DLS (solution-phase hydrodynamic diameter). All particles retained well-defined, spherical morphology consistent with the expected size and shape of icosahedral BMV particles, confirming successful *in vitro* assembly. (D) Encapsulation efficiency EE% of siRNA into VLPs at different siRNA:rCP mass ratios (1:4.8, 1:6, 1:8), whereas that encapsulated eGFP-siRNA was quantified using RiboGreen assay. The 1:4.8 ratio yielded the highest EE% (∼45 %), indicating that moderate siRNA excess enhances encapsulation. Bars represent the detectable siRNA content before VLPs destruction (washed/filtered) vs. post VLPs destruction (Released siRNA), with calculated EE% overlaid. Error bars indicate mean ± SD; p < 0.0001 is represented by (∗∗∗∗). (For interpretation of the references to color in this figure legend, the reader is referred to the Web version of this article.)

DLS measurements were performed on purified *wt*BMV, dissociated native CP, purified rCP, and RNA-loaded VLPs. Purified *wt*BMV exhibited a hydrodynamic diameter of approximately 30 nm with low polydispersity, indicating a homogeneous particle population (Fig. 1A). Dissociated capsid proteins and rCP showed smaller, slightly more heterogeneous size distributions; PDI ∼3.2 and ∼3.8, respectively, with particle sizes within the 5–7 nm range. This size range is consistent with the expected dimensions of BMV coat protein monomers and dimers in solution, as previously described elsewhere [8].

Interestingly, we observed that the hydrodynamic diameter of the assembled VLPs varied depending on the encapsulated RNA cargo used. VLPs assembled with yeast tRNA exhibited mean diameters comparable to *wt*BMV, ∼32 nm, whereas those formed with short synthetic siRNAs targeting eGFP and PD-L1 were notably smaller, averaging ∼20 nm in diameter (Fig. 1B). This variation likely arises from differences in the length, conformation, and charge distribution of the RNA templates. Yeast tRNA is approximately 76 nucleotides long and adopts a rigid, L-shaped tertiary structure stabilized by intramolecular base pairing, which provides a more defined scaffold for capsid assembly [38]. In contrast, siRNAs are short duplexes (∼21 nucleotides) with limited secondary structure and lower overall charge density. These characteristics may reduce their ability to efficiently initiate and sustain capsid assembly, thereby favoring the formation of smaller particles rather than complete icosahedral shells. Similar observations have been reported in the literature, where RNA length and structure directly influence the size and uniformity of plant virus-based VLPs [8,34]. It is also likely that the cargo type itself plays a significant role in modulating assembly, as the native genomic RNAs of BMV differ substantially in size, structure, and sequence-specific packaging signals compared to these heterologous RNAs used *in vitro* [7,8,34,[39], [40], [41]].

To further compare the morphology and structural integrity of both *wt*BMV and recombinant VLPs, we employed negative-stain TEM. Representative TEM images (Fig. 1C) confirmed that both *wt*BMV and RNA-loaded VLPs retained well-defined, spherical morphology consistent with icosahedral symmetry. The *wt*BMV particles exhibited uniform size distribution with an average diameter of ∼28–30 nm, consistent with DLS data. Similarly, VLPs encapsulating yeast tRNA displayed comparable dimensions (∼30–32 nm) in TEM, whereas those loaded with siRNAs targeting eGFP or PD-L1 were observed at approximately ∼30 nm in TEM, even though they appeared smaller in DLS ∼20 nm. This apparent discrepancy likely arises from methodological and imaging factors, TEM provides direct visualization of dehydrated particles, while DLS measures the hydrodynamic diameter in solution and may underestimate the size of weakly scattering, low-density siRNA-loaded VLPs [42]. Additionally, variations in magnification applied during TEM imaging to enhance particle visibility can minimize apparent size contrasts, even when scale bars remain identical across panels.

Nevertheless, these results align with prior reports demonstrating that the properties of encapsulated cargo, particularly length, folding, and charge distribution, can influence capsid morphology and compactness [43]. For instance, Strugała et al. (2021) observed that BMV recombinant VLPs assembled around different nucleic acid or polyanionic cargos exhibited distinct diameters, with more compact structures forming when shorter or denser cargos were encapsulated [8].

Collectively, the observed VLPs demonstrated minimal aggregation and preserved spherical architecture, indicating successful and stable *in vitro* assembly. Hence, the clarity and uniformity of the particles observed via TEM underscore the suitability of BMV VLPs for therapeutic RNA encapsulation, providing a structurally consistent platform for RNA delivery.

### 2.3 Encapsulation efficiency of siRNA into BMV-VLPs

Next, we evaluated the siRNA loading capacity of BMV-VLPs. Hence, we quantified the encapsulation efficiency (EE%) of the eGFP-siRNA as a model cargo. Thus, siRNA was co-incubated with rCP under controlled pH and ionic conditions to achieve self-assembly. Then, following VLPs disruption, released siRNA was quantified using the Quant-iT™ RiboGreen™ RNA assay, and EE% was calculated as the ratio of the released RNA to the initial input of RNA. To achieve siRNA encapsulation we firstly, used a CP:RNA mass ratio of 6:1, a commonly reported condition for BMV or other plant VLP encapsulation protocols, *e.g.,* Cowpea chlorotic mottle virus (CCMV), a closely related virus to BMV [7,44]. However, we noticed that this ratio yielded suboptimal encapsulation EE% of siRNA comparing to other ratios.

This low EE% was likely attributed to the short length (∼21 nt) and limited secondary structure of siRNA duplexes, which lack the complex folding and compact tertiary architecture characteristic of longer RNAs such as tRNA or viral genomic RNA. In natural BMV infection within a plant host, efficient encapsulation is facilitated by the presence of structured RNA packaging signals that promote strong electrostatic and structural interactions with the capsid interior [39]. In contrast, siRNA lacks such defined packaging motifs or tertiary folds, reducing its ability to effectively initiate and guide the cooperative assembly of CP monomers into an icosahedral particle.

To address this, we tested various CP:RNA ratios to evaluate whether those changes could improve RNA encapsulation. Although several ratios were screened, we report here the results from three representative conditions, *i.e.,* 4.8:1, 6:1 and 8:1 CP:RNA ratios. Among these conditions, the 4.8:1 ratio yielded the highest encapsulation efficiency (∼45 % ± 3.5), indicating that relative excess of siRNA improves loading efficiency, possibly by facilitating cooperative interactions during VLP assembly (Fig. 1D). Therefore, this 4.8:1 ratio was used for all subsequent siRNA delivery experiments.

The ∼45 % value reflects the siRNA recovered after downstream purification steps (centrifugation, filtration, and removal of aggregates), which introduce losses unrelated to the intrinsic encapsulation process. To determine the true encapsulation efficiency of VLP assembly, we quantified the siRNA content immediately after dialysis and prior to any purification. Under these conditions, disruptive sonication resulted in a 93 % increase in detectable siRNA, indicating that most of the input siRNA was successfully packaged during assembly.

Therefore, the intrinsic encapsulation efficiency of the VLP assembly process is ∼93 %, whereas the ∼45 % value represents the post-processing recovery yield. This lower recovery is consistent with the typical RNA and protein losses that occur during purification workflows involving centrifugation and filtration in nanoparticle and virus-like particle systems.

Overall, these findings are consistent with previous reports demonstrating that the structure, length, and charge density of the RNA cargo critically influence the size, uniformity, and loading capacity of plant VLPs. These outcomes further underscore the need to empirically optimize assembly conditions when adapting viral platforms to encapsulate non-native or synthetic RNA cargos [7,8,34,41].

### 2.4 Cytotoxicity assessment of BMV particles

A key prerequisite for the biomedical application of VLPs is their biocompatibility with target mammalian cells. To ensure the safety of BMV-VLPs intended for siRNA delivery and immunomodulation, we evaluated their potential cytotoxic effects in B16F10 murine melanoma cells. This cell line was chosen for its relevance as a melanoma model and also because it is used in the subsequent cellular uptake and gene silencing experiments, making toxicity assessment necessary for validating downstream efficacy studies. Hence, the biosafety evaluation in this work was limited to that cell type used in the delivery and immunomodulation assays. The assessment was conducted using native *wt*BMV particles, purified from infected plant tissue.

**Fig. 2:**
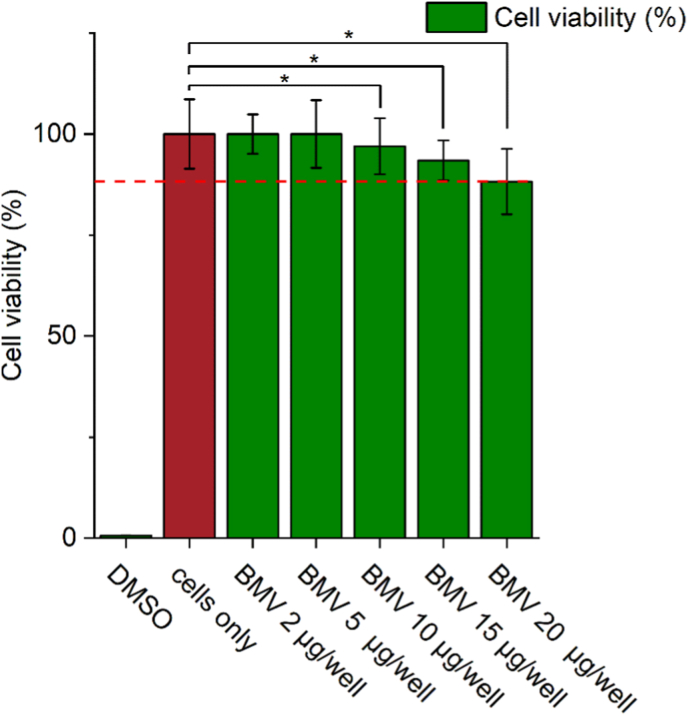
Cytotoxicity assessment of *wt*BMV particles in B16F10 melanoma cells. Cells were incubated with increasing concentrations of purified *wt*BMV (2, 5, 10, 15, and 20 μg per well; corresponding *to ∼2.62 × 10*^*6*^*, 6.55×10*^*6*^*, 1.31 × 10*^*7*^*, 1.97 × 10*^*7*^*and 2.62 × 10*^*7*^ BMV viral particles per cell, respectively) for 24 h. Cell viability was measured using the CellTiter® metabolic assay and compared to untreated controls and DMSO-treated cells. The virus concentration was standardized via UV absorbance at 260 nm using the BMV extinction coefficient *ε* = 5.15 cm^−1^ mg^−1^·mL and estimated particle numbers calculated using the known molecular weight of BMV (4.6 × 10^6^ g/mol) and Avogadro's number, yielding ∼1.31 × 10^11^ particles/μg. Results show no statistically significant cytotoxicity at lower doses (2 and 5 μg per well), while a statistically significant reduction in viability was observed at higher concentrations (≥10 μg per well), with all values remaining above 85 % viability. Error bars represent mean ± SD; ∗p < 0.05.

As shown in (Fig. 2), BMV particles exhibited no statistically significant cytotoxicity at lower concentrations (*i.e.,* 2 and 5 μg per well; corresponding to ∼2.62 × 10^6^ and 6.55 × 10^6^ BMV particles per cell, respectively), with cell viability remaining above 90 %. However, a statistically significant decrease in metabolic activity (∗*p* < 0.05) was observed starting from 10 μg per well and continued at 15 and 20 μg per well (*i.e., ∼*1.31 × 10^7^, 1.965 × 10^7^ and 2.62 × 10^7^ viral particles per cell, respectively), suggesting a mild but measurable dose-dependent cytotoxic effect. Nonetheless, even at the highest dose, cell viability remained above 85 %. Therefore, these results demonstrate that native *wt*BMV particles are largely biocompatible with B16F10 melanoma cells, exhibiting no statistically significant cytotoxicity at lower doses and only a statistically significant reduction in cell viability at higher concentrations (≥10 μg per well). Cell viability remained above 85 % across all treatment groups, and no morphological signs of cellular damage were observed. This indicates that BMV particles can be safely used at biologically relevant doses for siRNA delivery and other therapeutic applications, which support their development as a low toxicity nanocarrier platform for cancer immunotherapy. Likewise, these findings are consistent with previous studies demonstrating the high biocompatibility of BMV [7] and other plant virus nanoparticles across various mammalian cell lines, including CCMV [7,45], Cowpea mosaic virus **(**CPMV) [46], Physalis mottle virus (PhMV) [47] and TMV [48], where all of which reported minimal to no cytotoxic effects at comparable concentrations.

### 2.5 Cellular uptake of BMV nanoparticles

Efficient intracellular delivery is essential for the therapeutic efficacy of nanocarriers, particularly in the context of gene modulation strategies where the siRNA cargo must reach the cytoplasm to exert its function. Thus, to evaluate the ability of native *wt*BMV particles to be internalized by tumor cells, we conducted a comprehensive uptake study using the B16F10 murine melanoma cell line. As mentioned above, this cell model was selected for its direct relevance to downstream gene silencing and immunotherapy applications of melanoma [48,49].

Thus, to assess the interaction of those BMV particles with B16F10 cells, and the uptake potential of those particles, we applied a dual approach combining confocal microscopy, to visualize and spatially resolve the intracellular distribution of fluorescently labeled particles, and flow cytometry, to quantitatively analyze uptake across cell populations. Furthermore, to differentiate between surface-bound and truly internalized particles, we incorporated trypan blue quenching, a commonly used technique that allows discrimination of external fluorescence by selectively extinguishing non-internalized dye signals [50]. This multi-modal analysis enabled us to confirm the intracellular uptake of BMV particles and validate their localization within the cells, laying the foundation for their application in therapeutic siRNA delivery to melanoma cells.

**Fig. 3:**
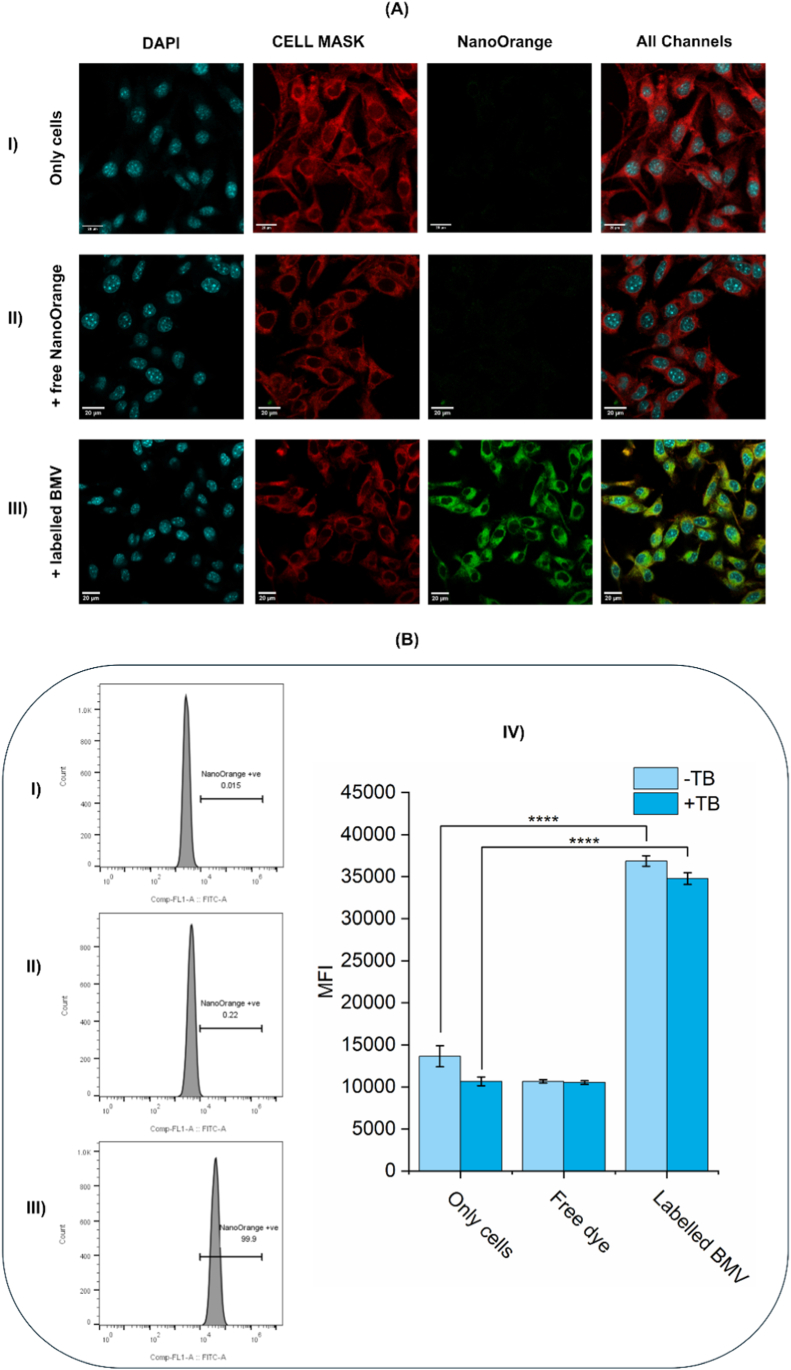
Intracellular uptake of NanoOrange-labeled BMV particles visualized by confocal microscopy and confirmed by flow cytometry. (A) Confocal images of B16F10 cells stained with DAPI (nuclei, blue) and CellMask™ (plasma membrane, red), showing NanoOrange signal (green) in: (I) untreated cells, (II) cells treated with free NanoOrange dye, and (III) cells treated with NanoOrange-labeled BMV particles. Robust cytoplasmic fluorescence in (III), distinct from nuclear and membrane markers, suggests the internalization of BMV particles. (B) Flow cytometry analysis of NanoOrange signal in (I) untreated cells, (II) cells with free dye, and (III) cells with labeled BMV. Fluorescence gated histograms show a distinct rightward shift in the BMV-treated group. (IV) Quantification of MFI ± trypan blue (TB) quenching confirms intracellular localization: Even after TB quenching, the signal in the BMV group remained significantly high in comparison to other control groups, verifying the BMV cellular internalization. ∗∗∗∗p < 0.0001. (For interpretation of the references to color in this figure legend, the reader is referred to the Web version of this article.)

Hereafter, to investigate the cellular internalization of BMV particles in melanoma cells, B16F10 cells were incubated with fluorescent dye NanoOrange®-labeled BMV virions [7], and uptake was visualized using confocal laser scanning microscopy. As shown in (Fig. 3A), untreated control cells (Fig. 3A–I) and cells treated with free dye (Fig. 3A–II) showed negligible background signals. Free NanoOrange exhibits negligible fluorescence in solution because it fluoresces strongly only upon protein binding [51], explaining the minimal signal in the free-dye control. Nonetheless, cells treated with labeled BMV particles exhibited strong green fluorescence (NanoOrange signal) in the cytoplasmic region (Fig. 3A–III). The merged images across all channels confirmed that the fluorescence was not associated with the plasma membrane or nuclei but rather distributed throughout the cytoplasm.

For quantitative analysis of the cellular uptake and to further confirm that the observed NanoOrange fluorescence signal originated from within the cells rather than from surface-bound particles, we performed flow cytometry analysis following the trypan blue quenching assay.

As shown in (Fig. 3B–I–III), representative flow cytometry histograms illustrate a clear shift in fluorescence intensity only in cells treated with NanoOrange-labeled comparing to the untreated or free dye groups. The fluorescence band in the labeled BMV group remained substantially above background even after trypan blue treatment, indicating that the fluorescent signal was protected from quenching. This intracellular signal was further quantified in (Fig. 3B–IV), where the mean fluorescence intensity (MFI) remained statistically significantly higher than the other control samples, untreated cells and the cells treated with free dye, in both trypan blue-treated and untreated groups. The persistence of high fluorescence intensity after trypan blue treatment in the labeled BMV group strongly supports that the signal originates from internalized virus particles, not merely membrane-associated ones. These findings are consistent with the confocal microscopy results and collectively confirm that BMV nanoparticles are effectively internalized by B16F10 cells.

**Fig. 4:**
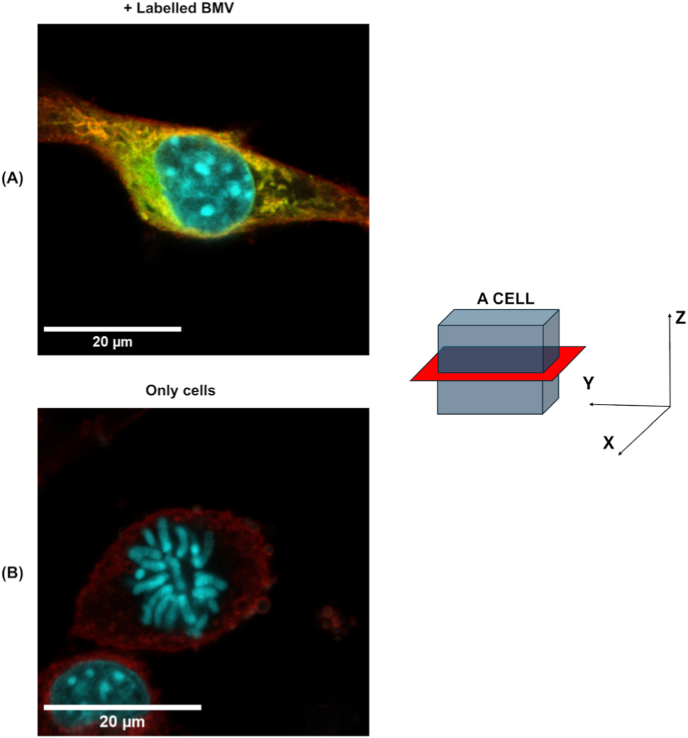
Z-stack confocal microscopy confirms intracellular localization of labeled BMV particles. (A) A representative *Z*-stack slice of a B16F10 cell treated with NanoOrange-labeled BMV particles, showing strong green/yellow fluorescence within the cytoplasm surrounding the nucleus (blue, DAPI), distinct from the plasma membrane (red, CellMask™). (B) Control image of untreated cells, showing no NanoOrange signal. The schematic on the right illustrates the orientation of the imaging plane (red slice) within the XYZ axes of the cell volume, highlighting how Z-stack acquisition enables optical sectioning at defined depths through the cell to confirm internalization. (For interpretation of the references to color in this figure legend, the reader is referred to the Web version of this article.)

The *Z*-stack intracellular image in (Fig. 4 and S4) further showed that cells treated with labeled BMV particles (Fig. 4, Panel A) exhibited robust green/yellow NanoOrange signal that colocalized within the cytoplasm but remained spatially distinct from the nucleus and plasma membrane signals, confirming internal localization. Untreated control cells (Fig. 4B) showed no visible NanoOrange signal. The schematic alongside the images illustrates the *Z*-axis acquisition plane, indicating how the red scanning layer was visualized to present subcellular distribution of BMV. Additionally, a video compiled from the Z-stack slices (Supplementary Video S1) visually highlights the fluorescence distribution throughout the cell volume and further emphasizes the marked difference between treated and untreated cells.

Although cellular internalization of BMV-VLPs was clearly demonstrated by confocal microscopy and flow cytometry, the specific endocytic pathways involved were not dissected in the present study; future work employing pharmacological inhibitors of clathrin- and caveolae-mediated uptake will be important to outline the dominant internalization mechanisms and their contribution to functional siRNA delivery.

### 2.6 Gene silencing efficiency and subsequent immunomodulation

#### 2.6.1 Gene silencing efficiency

RNA interference (RNAi) is a post-transcriptional gene regulation mechanism in which siRNA guide the selective degradation of complementary mRNA molecules via the RNA-induced silencing complex (RISC) [27]. The high sequence specificity and modularity of RNAi make siRNAs promising tools for targeted modulation of disease-associated genes [24,37,52,53]. However, their clinical translation remains challenging: siRNAs are unstable in biological fluids, rapidly degraded by nucleases, and inefficiently internalized by many mammalian cells. Moreover, endosomal entrapment frequently restricts siRNAs from reaching the cytoplasm, the site of RISC activity, limiting functional knockdown [29]. These limitations warrant effective delivery platforms capable of protecting siRNAs, facilitating uptake, and enabling cytoplasmic release. In this context, we evaluated whether recombinant BMV-VLPs could deliver functional siRNAs across diverse immunologically and oncologically relevant cell types.

**Fig. 5:**
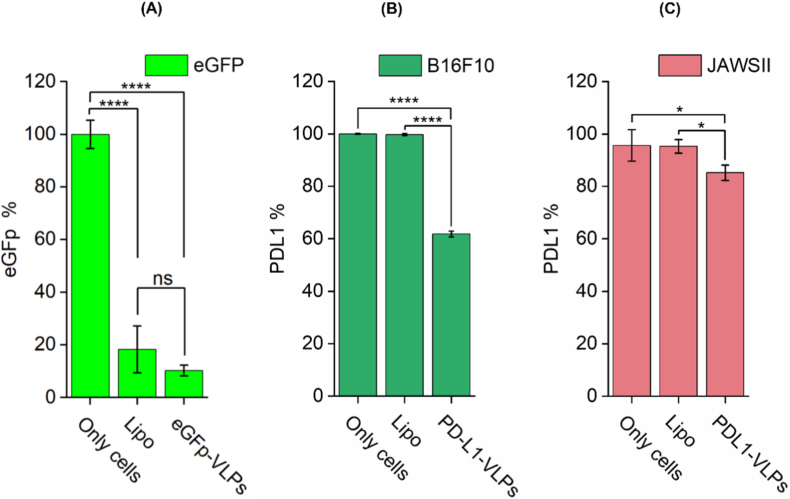
Gene silencing efficiency of BMV-VLPs delivering siRNA targeting eGFP and PD-L1, where untreated cells (Only cells) were used as controls. (A) eGFP knockdown in RAW264.7 cells following treatment with eGFP-siRNA-loaded BMV-VLPs (eGFP-VLPs) (final siRNA concentration ∼50 nM) or Lipofectamine RNAiMAX (Lipo) complexed with the same siRNA. Both treatments significantly reduced the percentage of eGFP-positive cells compared to untreated controls (∗∗∗∗p < 0.0001). Although, the difference between Lipo and eGFP-VLPs was not statistically significant (ns). (B) PD-L1 silencing in B16F10 melanoma cells treated with PD-L1-siRNA-loaded BMV-VLPs (PD-L1-VLP) or Lipofectamine-siRNA complexes, where PD-L1 surface expression was significantly reduced in cells treated with VLPs (∗∗∗∗p < 0.0001). (C) PD-L1 silencing in JAWSII dendritic cells shows a moderate but statistically significant decrease in PD-L1 expression following treatment with PDL1-VLPs compared to untreated and Lipo-treated controls (∗p < 0.05).

As an initial proof of concept, siRNA targeting enhanced eGFP was encapsulated within BMV-VLPs (eGFP-VLPs) and delivered to RAW264.7 macrophage-like cells stably expressing eGFP. Cells were treated twice (double transfection) at a final siRNA concentration of 50 nM, and fluorescence was quantified 72 h later by flow cytometry. As shown in (Fig. 5A), both eGFP-VLPs and Lipofectamine RNAiMAX significantly reduced the eGFP-positive population compared to untreated cells (∗∗p < 0.0001). The difference between Lipofectamine and eGFP-VLPs did not reach statistical significance (ns), reflecting the variability observed in the Lipofectamine-treated replicates. Fluorescence microscopy confirmed these findings (Fig. S5). These results demonstrate that BMV-VLPs can deliver functional siRNA into macrophage-lineage cells, which are often refractory to lipid-based transfection because of high nuclease content, phagocytic activity, and endosomal complexity [54].

While lipofectamine-based reagents are widely used *in vitro* for their transfection efficiency, their membrane-disruptive and cytotoxic properties significantly limit their applicability in *in vivo* and clinical settings [55]. In contrast, BMV-VLPs can achieve more effective gene silencing while exhibiting lower cytotoxicity than Lipofectamine, highlighting the potential of BMV-VLPs as a biocompatible and efficient platform for therapeutic siRNA delivery.

We next evaluated the ability of BMV-VLPs to silence PD-L1, a key immune checkpoint molecule central to tumor immune escape and T-cell suppression [21]. PD-L1 expression on melanoma cells and antigen-presenting cells (APCs) attenuates T-cell proliferation, cytokine production, and cytotoxic activity [21,56,57]. Clinical blockade of PD-1/PD-L1 interactions using monoclonal antibodies, such as pembrolizumab, nivolumab, atezolizumab, and others [25,26], has transformed melanoma treatment. However, these biologics can cause immune-related toxicities, showing variable patient response rates, and are limited by cost and resistance mechanisms [21,58]. On the contrary, siRNA-based knockdown of PD-L1 offers a mechanistically distinct strategy capable of intracellular modulation of PD-L1 expression with potentially reduced systemic toxicity.

To assess PD-L1 silencing, we treated two cell types relevant to melanoma immunology: B16F10 melanoma cells and JAWS II dendritic cells (DCs). B16F10 cells are widely used to model tumor immune evasion and upregulate PD-L1 in response to inflammatory cues [59,60]. JAWS II cells represent immature murine DCs that play essential roles in priming T-cell responses [61,62]; PD-L1 expression on DCs is a key mechanism by which tumors induce antigen-specific tolerance [52]. PD-L1-targeting siRNA was encapsulated using a 4.8:1 rCP:RNA ratio, previously identified as optimal for loading efficiency.

Following two sequential treatments (final siRNA concentration 50 nM), PD-L1 surface levels were measured by flow cytometry. In B16F10 cells, PDL1-VLPs reduced PD-L1 expression by approximately 50 % relative to untreated cells (Fig. 5B; ∗∗∗∗p < 0.0001) and performed more effectively than Lipofectamine RNAiMAX. Because PD-L1 directly contributes to melanoma-mediated suppression of cytotoxic T cells, this degree of knockdown strongly supports the potential utility of BMV-VLP–mediated RNAi in immune-checkpoint targeting.

In contrast, PD-L1 knockdown in JAWS II DCs was modest (∼15 % reduction; Fig. 5C; ∗p < 0.05). This difference likely reflects intrinsic features of this cell type: DCs possess robust endosomal processing pathways, increased nucleases, and activation-sensitive phenotypes that reduce siRNA cytoplasmic delivery and can impair RNAi efficiency [61,63]. Additionally, siRNA-induced stress responses in DCs can alter membrane trafficking or promote maturation, further complicating intracellular delivery [[56], [57], [58], [59]]. Although Lipofectamine RNAiMAX is widely used as a benchmark reagent, its functional silencing efficiency is known to be strongly cell-type and target-dependent, particularly for immune-lineage cells and inducible endogenous targets such as PD-L1 [64,65].

Despite this lower knockdown efficiency, the ability of BMV-VLPs to silence genes in both tumor cells and primary immune cells highlights their versatility. Results from RAW264.7, B16F10, and JAWS II cells collectively demonstrate that recombinant BMV-VLPs can deliver functional siRNA across multiple cell lineages crucial to cancer immunotherapy.

This study expands on previous work using plant-extracted BMV particles for siRNA delivery [7] by demonstrating that recombinant BMV capsid proteins can assemble VLPs capable of packaging and delivering functional siRNA in mammalian systems. The recombinant approach provides greater flexibility for engineering and avoids reliance on plant-derived material, enabling future surface modifications for targeted delivery. Furthermore, our findings reinforce the concept that VLP assembly behavior is influenced by RNA cargo structure and charge density, consistent with prior observations in plant-virus systems [8,66]. These features position recombinant BMV-VLPs as a robust platform for customizable RNA delivery in immunotherapy applications.

#### 2.6.2 Functional immunomodulation following PD-L1 silencing in melanoma cells

To determine whether PD-L1 knockdown translates into functional restoration of antitumor immunity, we evaluated the susceptibility of melanoma cells to antigen-specific T-cell cytotoxicity following treatment with PD-L1-siRNA-loaded BMV-VLPs (PDL1-VLPs). B16-OVA melanoma cells were treated twice with PDL1-VLPs, washed, and subsequently co-cultured with OT-I CD8^+^ T cells at a 1:1 effector-to-target ratio.

**Fig. 6:**
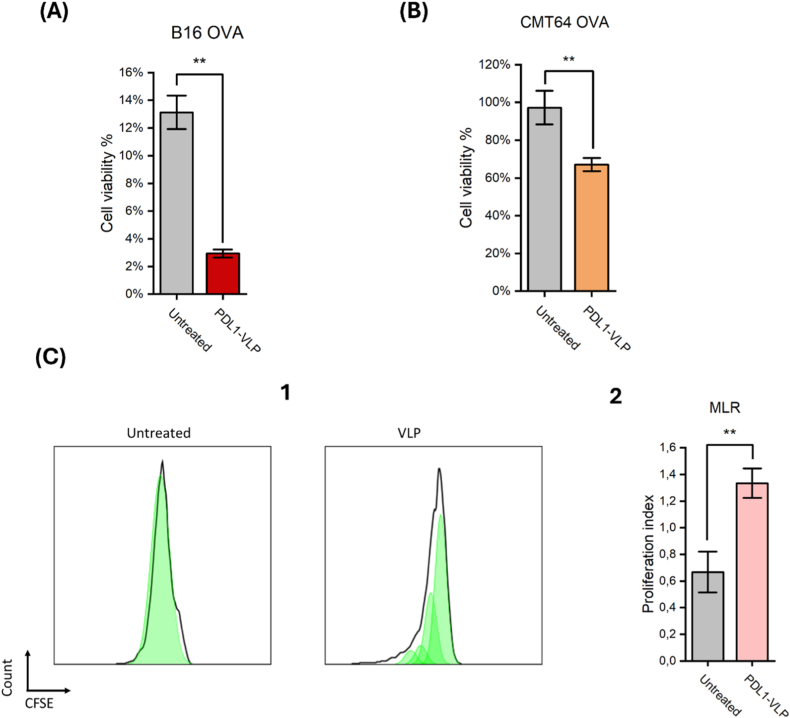
*Functional immunomodulation following PD-L1 silencing with BMV-VLPs.* (A) OT-I T-cell–mediated killing of B16-OVA melanoma cells following pretreatment with PDL1-VLPs. Melanoma viability decreased from ∼12 % in untreated cells to ∼3 % after PDL1-VLP treatment. (B) OT-I T-cell–mediated killing of CMT64-OVA cells under reduced effector pressure (10:1 tumor:T cell). PDL1-VLP pretreatment increased killing by >30 % compared to untreated cells. (C1) CFSE-based proliferation profiles from mixed leukocyte reactions (MLR). T cells co-cultured with untreated dendritic cells show minimal CFSE dilution, whereas PDL1-VLP–treated dendritic cells induce multiple dilution peaks. (C2) Quantification of T-cell proliferation in MLR cultures, showing a significantly higher proliferation index in co-cultures with PDL1-VLP–treated dendritic cells compared to untreated controls (p < 0.01).

As shown in (Fig. 6A), OT-I T cells reduced the viability of untreated B16-OVA cells to ∼12 %, consistent with the strong immunogenicity of the OVA antigen. Notably, pretreatment with PDL1-VLPs further sensitized melanoma cells to T-cell–mediated killing, reducing viability to ∼3 %. This amplified loss of viable tumor cells reflects the functional effect of PD-L1 suppression, which removes inhibitory PD-1/PD-L1 signals at the tumor–T cell interface and restores cytotoxic T-cell activity. These results provide direct evidence that siRNA-mediated PD-L1 knockdown enhances melanoma cell vulnerability to T-cell attack, supporting the relevance of BMV-VLP–based RNAi for immune-checkpoint modulation.

#### 2.6.3 Functional validation in a secondary tumor model (CMT64-OVA)

To further determine whether the immunomodulatory effect of PD-L1 silencing extends beyond melanoma, we performed a secondary validation using CMT64-OVA cells, a poorly immunogenic murine lung carcinoma line known to exhibit reduced susceptibility to cytotoxic lymphocytes [67,68]. Because strong killing in preliminary experiments masked differences between treatments, the assay was performed under reduced T-cell pressure using a 10:1 tumor-to-T-cell ratio.

As shown in (Fig. 6B), OT-I T cells induced only modest killing of untreated CMT64-OVA cells under these low-effector conditions, consistent with the immune-resistant nature of this tumor model. Importantly, pretreatment with PDL1-VLPs increased OT-I–mediated killing by more than 30 % compared to untreated controls. Although the magnitude of killing was lower than in B16-OVA cells, this is expected given the reduced effector-to-target ratio and lower baseline immunogenicity of CMT64. These results confirm that the functional benefits of PD-L1 knockdown, enhanced susceptibility to antigen-specific T-cell cytotoxicity, are preserved across tumor models with distinct immune sensitivities.

#### 2.6.4 Mixed leukocyte reaction (MLR): functional immunomodulation in human APC–T cell interactions

Finally, we wanted to investigate the functional effects of PD-L1 silencing in a human immune setting. So, we employed a mixed-lymphocyte reaction (MLR), a classical assay in which APCs from one donor stimulate allogeneic T cells from another. First described in early immunogenetic studies [66], the MLR remains a widely used model for dissecting APC–T cell communication. Mechanistic work has shown that dendritic cells drive T-cell proliferation in MLR cultures through coordinated antigen presentation, costimulatory signaling, and regulatory inputs [69]. Among these pathways, PD-L1 expressed on APCs serves as an important inhibitory signal that restrains allogeneic T-cell proliferation; blocking this interaction markedly enhances T-cell activation in MLR assays [70].

In our experiments, monocytes from donor **A** were differentiated into dendritic cells and treated with two sequential doses of PDL1-VLPs before co-culture with allogeneic T cells from donor **B**. T-cell proliferation was quantified using CFSE labeling, where successive cell divisions produce characteristic fluorescence-dilution peaks.

As shown in (Fig. 6, Fig. 2), T cells co-cultured with untreated dendritic cells retained a single undiluted CFSE peak, indicating minimal proliferation. In contrast, dendritic cells pretreated with PDL1-VLPs induced multiple CFSE dilution peaks, reflecting substantially increased T-cell expansion. This enhanced proliferation aligns with the expected consequences of reducing PD-L1–mediated inhibition on dendritic cells, thereby strengthening allogeneic T-cell activation.

Together, these data demonstrate that PDL1-VLP–mediated RNAi effectively modulates human APC–T cell interactions, restoring T-cell activation in a physiologically relevant assay. When integrated with the functional killing results in B16-OVA and CMT64-OVA cells, the MLR findings confirm that PD-L1 knockdown produces coherent immunological effects across tumor and immune-cell contexts, highlighting the translational potential of recombinant BMV-VLPs as a platform for RNAi-based checkpoint modulation.

We noted that despite variations in silencing efficiency across cell types, the versatility of BMV-VLPs provides a strong foundation for future optimization. As with other plant-virus-based delivery systems, a key limitation is the absence of inherent cell-type specificity [17,46]. Further engineering, for example, through receptor-targeting ligands or peptide modifications [19], may allow more selective delivery to tumor or immune cell subsets.

As this work was designed as an *in vitro* evaluation of recombinant BMV-VLPs, several limitations should be noted. The biosafety assessment performed here was restricted to the same cell types used in the delivery and immunomodulation assays, and broader toxicity evaluation in non-malignant cells was not conducted. Likewise, *wt*BMV particles were used only in preliminary uptake and all functional delivery experiments employed recombinant BMV-VLPs. A systematic comparison between these formulations, including serum stability and siRNA release kinetics, was beyond the scope of this study. Likewise, direct benchmarking against commercial LNPs systems or other plant virus–derived VLP platforms was not included here and shall be explored in future work. Nevertheless, our results demonstrate that BMV-VLPs can efficiently encapsulate and deliver functional siRNA and modulate immune-checkpoint pathways across multiple biologically relevant cell types involved in cancer immunotherapy.

## 3 Conclusions

In this study, we establish recombinant BMV-VLPs as a versatile and biocompatible platform for siRNA delivery with clear potential for melanoma immunotherapy. Using recombinant capsid proteins, we efficiently encapsulated siRNA and achieved robust gene silencing across multiple mammalian cell types, including B16F10 melanoma cells, JAWS II dendritic cells, and RAW264.7 macrophage-like cells. Notably, BMV-VLPs effectively downregulated PD-L1 expression in both tumor cells and antigen-presenting cells, two central regulators of immune escape, highlighting the relevance of this platform for immune-checkpoint modulation. Although the extent of PD-L1 silencing varied across cell types, knockdown was consistently detectable and, in most cases, exceeded that achieved with conventional lipid-based delivery systems.

Beyond checkpoint suppression, functional assays demonstrated that PD-L1 silencing using BMV-VLPs restored T-cell cytotoxicity in melanoma cells, sensitized a secondary tumor model (CMT64-OVA) to antigen-specific killing, and enhanced allogeneic T-cell proliferation in a mixed leukocyte reaction. These complementary findings confirm that BMV-VLP–mediated RNAi produces coherent immunological effects in both tumor and immune-cell contexts, extending the utility of the platform beyond simple gene knockdown.

Together, these results position recombinant BMV-VLPs as a promising foundation for RNA-based cancer immunotherapy. Their low cytotoxicity, modular assembly, and ability to deliver functional siRNA into typically difficult-to-transfect immune cells underscore their translational potential. While this work focused on *in vitro* characterization, future studies should explore strategies to enhance cell-type specificity and elucidate uptake and trafficking pathways, which will be essential for advancing this delivery system toward targeted *in vivo* applications and for shaping immune responses within the tumor microenvironment.

## 4 Materials and methods

### 4.1 Production and purification of wild-type brome mosaic virus *wt*BMV

The *wt*BMV particles were propagated in *Hordeum vulgare* (barley) plants as previously described [7,71,72]. Briefly, barley seeds were sown and grown in a controlled growth chamber at 22 °C under a 16 h light/8 h dark photoperiod. Fourteen-day old seedlings were mechanically inoculated, using a homogenate of infected leaves obtained from DSMZ (Braunschweig, Germany; Cat. No. PC-0178) as the inoculum source, by rubbing the leaves with carborundum and then adding the virus extract suspended inoculation buffer (0.01M sodium phosphate, 0.01 M magnesium chloride, pH 6). Inoculated plants were maintained under the same conditions and harvested two weeks after inoculation, where only those showing clear mosaic symptoms were selected for virus extraction and stored in −80 °C.

Frozen infected leaves were homogenized in cold extraction buffer (0.5 M sodium acetate, 0.3 M boric acid, 0.01 M magnesium chloride, pH 4.5), 4 mL per 1 g of tissue. The homogenate was then filtered through double-layered cheesecloth to remove debris, and the flowthrough was mixed with one-fifth volume of chloroform by vortexing for 30 s at 2500 rpm. The mixture was clarified by centrifugation at 5000×*g* for 5 min at 4 °C. The upper aqueous phase was carefully transferred to fresh tubes, and PEG 8000 (Sigma-Aldrich) was added to final concentration of ∼8 % to precipitate the virus particles. The supernatant-PEG mixture was incubated on ice for 30 min and centrifuged again at 12,000×*g* for 15 min at 4 °C. The supernatant was discarded, and the resulting pellet was resuspended in 2 mL of cold phosphate storage buffer (0.02 M sodium phosphate, pH 7.5) and incubated overnight at 4 °C with gentle shaking (750 rpm) to allow complete dissolution. The virus suspension was then layered onto a 10 % (w/v) sucrose cushion and centrifuged at 150,000×*g* for 2 h at 4 °C using a Beckman SW 32 Ti Swinging-Bucket Rotor. The pellet was resuspended again in phosphate buffer and further purified by ultracentrifugation on a 10–40 % (w/v) continuous sucrose gradient prepared in the same buffer at 110,000×*g* for 2 h at 4 °C. The visible virus band was collected, diluted in phosphate buffer, and concentrated by ultrafiltration using Amicon Ultra centrifugal filters (100 kDa MWCO, Millipore). The final virus preparation was resuspended in virus storage buffer (0.5 M sodium acetate and 0.08 M magnesium acetate, pH 4.5). and stored at −80 °C. The final virus purity was confirmed by SDS-PAGE, and concentration was determined by measuring UV absorbance at 260 nm using a NanoDrop 2000c spectrophotometer (Thermo Fisher Scientific), applying the BMV extinction coefficient *ε* = 5.15 cm^−1^ mg^−1^ mL. This *ε* value reflects the combined absorbance at 260 nm of both the RNA and protein components present in the intact virion. The number of virus particles per unit mass was then estimated using the molecular weight of a single BMV particle (MW ≈ 4.6 × 10^6^ g/mol) [73] and the Avogadro's number (NA = 6.022 × 10^23^ mol^−1^), according to Equation:

$$Particles\;per\;\mu g=\frac{N_{A}}{MW}\times 10^{-6}\approx 1.31\times 10^{11}\;particles/\mu g$$

This method provides an approximate and reproducible estimate of particle concentration based on spectrophotometric data, assuming the virus is intact and free from significant protein or RNA contaminants [74].

### 4.2 Recombinant BMV capsid protein expression and VLP assembly

The BMV CP was recombinantly expressed in *E. coli* BL21 (DE3) pLysS using the pMCSG48 vector system. The plasmid was a generous gift from Prof. Anna Urbanowicz (Polish Academy of Sciences, Poznan, Poland), where the BMV CP sequence (GenBank accession: KP096132.1) was cloned into the pMCSG48 plasmid following the ligation-independent cloning (LIC) protocol. This vector encodes a NusA-His6-TEV fusion upstream of the target gene, enabling better soluble expression and affinity purification. The final protein product corresponds to the mature BMV CP with the following amino acid sequence:

SNIMSTSGTGKMTRAQRRAAARRNRRTAGVQPVIVEPIAAGQGKAIKAIAGYSISKWEASSDAITAKATNAMSITLPHELSSEKNKELKVGRVLLWLGLLPSVAGRIKACVAEKQAQAEAAFQVALAVADSSKEVVAAMYTDAFRGATLGDLLNLQIYLYASEAVPAKAVVVHLEVEHVRPTFDDFFTPVYK.

Transformed *E. coli* BL21 (DE3) pLysS cells were cultured in LB medium containing 100 mg/L ampicillin and 34 mg/L chloramphenicol at 37 °C until reaching an OD_600_ of 0.6–0.8, at which point expression was induced with 0.5 mM IPTG. The culture was incubated overnight at 20 °C with shaking (200 rpm). Cells were harvested by centrifugation at 5000×*g* for 20 min at 4 °C, and the pellet was stored at −20 °C until purification.

Cell pellets were resuspended in lysis buffer (25 mM Tris-HCl pH 8, 500 mM NaCl, 0.2 mg/mL lysozyme (sigmaAldrich), 250 U benzonase nuclease (sigmaAldrich)), then lysed by EmulsiFlex high-pressure homogenizer (Avestin). The lysate was clarified by centrifugation at 20,000×*g* for 30 min at 4 °C. The supernatant was filtered through 0.22 μm filters (Sigma-Aldrich) and mixed with of same volume of buffer II (25 mM Tris-HCl pH 8, 500 mM NaCl, 20 mM imidazole (Sigma-Aldrich) and loaded onto a Ni-NTA affinity column (HisTrap HP, Cytiva) pre-equilibrated with buffer II. Bound proteins were washed with buffer II and eluted with 250 mM imidazole in buffer II [8]. The eluted protein was then dialyzed overnight using buffer III (25 mM Tris-HCl pH 8, 0.5 M NaCl) to remove imidazole.

To remove the NusA fusion tag, the eluted protein was incubated overnight at 4 °C with in-house purified TEV protease (produced according to Kapust et al., [75] Addgene plasmid pRK793, #8827) at a protease:substrate ratio of 1:50 (w/w). The cleaved mixture was reloaded onto a Ni-NTA column to separate the His-tagged NusA and TEV protease from the His-tag-free BMV CP, which was then collected in the flow-through. The flowthrough was then dialyzed in protein storage buffer (PSB); (1 M NaCl, 1 mM EDTA, 20 mM Tris-HCl, pH 7.2). The final purified rCP was concentrated by ultrafiltration using Amicon Ultra centrifugal filters (10 kDa MWCO, Millipore) and its purity was evaluated by SDS-PAGE, as well as the monodispersity was confirmed by DLS measurements using a Zetasizer Nano ZS (Malvern Panalytical, GB). The concentration of the BMV coat proteins was determined by measuring the absorbance at 280 nm, where the calculated BMV rCP *ε* = 18450 M^−1^cm^−1^. Additionally, the concentration was also confirmed using bicinchoninic acid (BCA) protein assay (Pierce™ BCA Protein Assay Kit, Thermo Fisher Scientific). The purified protein was eventually mixed with glycerol to a final concentration of 15 % (v/v) as a cryoprotectant, flash-frozen in liquid nitrogen, and stored at −80 °C.

### 4.3 VLP assembly and siRNA encapsulation efficiency

BMV-VLPs were assembled *in vitro* by mixing purified rCP with either yeast tRNA (Thermo Fisher Scientific) or synthetic siRNA cargo at defined rCP:RNA mass ratios. A 6:1 ratio was used for tRNA-based VLP formation, while siRNA was assembled at ratios ranging from 4:1 to 8:1. Based on encapsulation performance, a 4.8:1 ratio was selected for subsequent experiments. Assembly was carried out in two sequential buffer conditions. The rCP and RNA were first incubated in assembly buffer I (50 mM Tris-HCl, pH 7.2, 50 mM NaCl, 10 mM KCl, 5 mM MgCl_2_, 1 mM Dithiothreitol (DTT)) and then dialyzed into virus storage buffer (50 mM sodium acetate, pH 4.5, 8 mM magnesium acetate) at 4 °C to promote particle formation and stabilization. Assembled VLPs were purified by ultrafiltration using 100 kDa Amicon Ultra centrifugal filters (Millipore) at 4700×*g* for 15 min at 4 °C and washed three times with virus storage buffer to remove unencapsulated RNA and excess protein.

To evaluate siRNA encapsulation efficiency, VLPs were treated with RNase A (10 μg/mL, 30 min at 37 °C) to digest any unencapsulated siRNA. The samples were then treated with Proteinase K treatment (200 μg/mL, 30 min at 55 °C) followed by 0.1 % SDS denaturation. The particles were subsequently disrupted by sonication on ice (3 × 10 s pulses with 20 s rest intervals). Released siRNA was quantified using the Quant-iT™ RiboGreen™ RNA assay (Thermo Fisher Scientific), and encapsulation efficiency was calculated as the percentage of recovered RNA relative to the initial RNA input [66].

The siRNA duplex targeting enhanced eGFP was custom-synthesized by Eurogentec (Belgium) based on the eGFP coding sequence expressed in RAW 264.7 cells (Cellomics Technology).

The siRNA duplex was.

• **Sense strand**: 5′-CAA GCU GAC CCU GAA GUU C-3′
• **Antisense strand**: 5′-GAA CUU CAG GGU CAG CUU G-3′

### 4.4 Biochemical characterization: SDS-PAGE and ELISA

The purity of the BMV CP was assessed by SDS-PAGE. Samples were prepared by mixing with Novex™ Tris-Glycine SDS Sample Buffer (Thermo Fisher Scientific, Cat. No. LC2676) and heated at 95 °C for 10 min. Equal volumes of protein solutions were loaded onto 4–20 % Mini-PROTEAN® TGX™ precast polyacrylamide gels (Bio-Rad) and separated at 150 V for approximately 45 min. Following electrophoresis, gels were stained using Pierce™ Coomassie Brilliant Blue R-250 Staining Solution (Thermo Fisher Scientific, Cat. No. 24620) according to the manufacturer's protocol. Gels were imaged using a ChemiDoc™ MP imaging system (Bio-Rad) and analyzed with Image Lab software.

An antigen-specific ELISA was performed using the commercial BMV ELISA kit (Agdia, USA, Cat. No. SRA 29300). The assay was carried out following the manufacturer's instructions. Briefly, 100 μL of each sample or control was added to the pre-coated microtiter wells and incubated with the provided anti-BMV monoclonal antibody conjugated to alkaline phosphatase. After washing, the substrate was added and color development was monitored. Absorbance was measured at 405 nm using a microplate reader Varioskan™ LUX (Thermo Fisher Scientific). Each sample was analyzed in triplicate, and positive/negative controls provided in the kit were included for assay validation.

### 4.5 Biophysical characterization of BMV-VLPs: DLS and TEM

DLS and TEM were used to assess the size distribution and morphology of *wt*BMV assembled BMV-VLPs. For DLS, samples were prepared in virus storage buffer at a final protein concentration of approximately 0.2 mg/mL, as determined by BCA protein assay (Pierce™ BCA Protein Assay Kit, Thermo Fisher Scientific). Measurements were performed using a Zetasizer Nano ZS (Malvern Instruments) at 4 °C in low-volume quartz cuvettes. Each sample was measured in triplicate, and average hydrodynamic diameters and PDI values were calculated using the instrument's built-in software.

For TEM analysis, 5 μL of assembled VLPs was adsorbed onto carbon-coated 400-mesh copper grids (Electron Microscopy Sciences) for 1 min and excess liquid was removed by blotting with filter paper. Grids were then negatively stained with 2 % (w/v) uranyl acetate for 30 s and air-dried. Imaging was performed using a transmission electron microscope Hitachi HT7800 (Hitachi High-Tech, Tokyo, Japan) operated at 120 kV. Particle morphology and size were assessed using ImageJ-Fiji software.

### 4.6 Cytotoxicity assessment

The cytotoxicity of *wt*BMV was evaluated in B16F10 melanoma cells using the CellTiter® cell viability assay (Promega), which measures metabolic activity. B16F10 cells were seeded in 96-well plates at a density of 1 × 10^4^ cells per well in complete DMEM medium and allowed to adhere overnight at 37 °C in a 5 % CO_2_ incubator. Cells were then treated with *wt*BMV particles at doses of 2, 5, 10, 15, and 20 μg per well. Based on the estimated molecular weight of BMV (4.6 × 10^6^ g/mol) and an approximate particle number of 1.31 × 10^11^ virions per μg, these doses correspond to ∼2.62 × 10^11^ to 2.62 × 10^12^ particles per well, or ∼2.62 × 10^6^ to 2.62 × 10^7^ particles per cell.

After 24 h of incubation, cell viability was assessed by adding CellTiter® reagent to each well according to the manufacturer's instructions. Absorbance was measured using a microplate reader Varioskan™ LUX (Thermo Fisher Scientific). Untreated cells served as negative controls, while DMSO-treated cells were used as positive controls for cytotoxicity. Viability was expressed as a percentage relative to untreated cells, and experiments were performed in triplicate.

### 4.7 Cellular uptake

To evaluate cellular uptake of *wt*BMV particles, B16F10 melanoma cells were treated with NanoOrange®-labeled *wt*BMV. Labeling was performed using NanoOrange Protein Labeling Reagent (Thermo Fisher Scientific), following the manufacturer's protocol and the procedure described by Nuñez-Rivera et al. [7]. Unbound dye was removed by ultrafiltration using Amicon Ultra centrifugal filters (100 kDa MWCO, Millipore), and labeled particles were resuspended in phosphate-buffered saline (PBS, pH 7.4). The qualitative intracellular uptake of BMV particles was evaluated by confocal microscopy using Leica TCS SP8 confocal laser scanning microscope (Leica Microsystems, Germany).

Briefly, B16F10 cells were seeded at a density of 5 × 10^4^ cells per well in 8-well glass-bottom chambers (Lab-Tek II, Thermo Fisher Scientific) and allowed to adhere overnight. Cells were then incubated with NanoOrange-labeled *wt*BMV at a concentration equivalent to approximately 1.3 × 10^6^ virions per cell (10 μg per well) at 37 °C. After incubation, cells were washed twice with PBS and stained with CellMask™ Deep Red plasma membrane dye (Thermo Fisher Scientific) for 10 min at room temperature. Nuclei were counterstained with DAPI (2.5 μg/mL, Thermo Fisher Scientific), followed by three PBS washes.

Cells were fixed with 4 % (v/v) paraformaldehyde for 15 min at room temperature. Imaging was performed using a 63 × water-immersion objective, acquiring both single-plane and *Z*-stack images. Image analysis and three-dimensional reconstruction were carried out using Leica Application Suite (LAS) software and ImageJ-Fiji.

Quantitative analysis of BMV uptake in B16F10 cells was performed using flow cytometry under the same conditions described for confocal imaging. After 24 h of incubation with NanoOrange-labeled BMV particles, cells were washed with PBS and detached using 0.05 % trypsin-EDTA (Thermo Fisher Scientific). To quench extracellular fluorescence and assess internalization, cells were treated with trypan blue (0.005 % v/v) for 5 min on ice prior to acquisition. Confocal images were processed and analyzed using ImageJ–Fiji software, with contrast adjustment applied uniformly across groups.

Flow cytometric analysis was carried out using a BD Accuri™ C6 flow cytometer, recording at least 10,000 events per sample. Data analysis was performed using FlowJo software (BD Biosciences), and MFI was used to quantify internalized particles relative to untreated controls.

### 4.8 Gene silencing assays

Gene silencing efficiency was assessed in three murine cell models: RAW 264.7-GFP macrophages (stably expressing eGFP), B16F10 melanoma cells, and JAWS II immature dendritic cells. Cells were cultured according to the manufacturer's recommendations: RAW 264.7-GFP cells in RPMI 1640, and B16F10 and JAWS II in DMEM or α-MEM, respectively, all supplemented with 10 % (v/v) fetal bovine serum (FBS). Cells were maintained at 37 °C in a humidified incubator under 95 % air and 5 % CO_2_. Seeding densities were optimized for each cell line to reach ∼70–80 % confluency at the time of treatment, accounting for their respective growth rates (*e.g.,* B16F10 was seeded at lower density due to rapid proliferation).

BMV-VLPs loaded with siRNA targeting eGFP or PD-L1 were added directly to cells in serum-containing medium at a final siRNA concentration of 50 nM. Cells were treated twice (at 0 h and 24 h). Following each incubation with VLPs, the suspension was removed and replaced with fresh medium. Lipofectamine™ RNAiMAX (Thermo Fisher Scientific) was used as a positive control at the siRNA-to-reagent ratio recommended by the manufacturer. Untreated cells served as negative controls.

At 72 h post-initial treatment, cells were harvested using 0.05 % trypsin-EDTA, washed with PBS, and analyzed by flow cytometry (BD Accuri™ C6). eGFP expression in RAW 264.7-GFP cells was measured in the FITC channel (excitation/emission ∼488/530 nm), while PD-L1 expression in B16F10 and JAWS II cells was detected by immunostaining with FITC-conjugated anti-mouse PD-L1 antibody (BioLegend). At least 10,000 events were collected per sample, and data were analyzed using FlowJo software. Knockdown efficiency was expressed as the percentage reduction in MFI relative to untreated controls. In parallel, fluorescence microscopy was also used to visually assess eGFP silencing in RAW 264.7-GFP cells, using an EVOS M5000 Imaging System (Thermo Fisher Scientific) equipped with appropriate filter sets for GFP. Fluorescence intensity was qualitatively compared between untreated controls and cells treated with Lipofectamine-eGFP-siRNA complex or eGFP-VLPs.

### 4.9 Antigen-specific T-cell cytotoxicity assay

B16-OVA melanoma cells and CMT64-OVA carcinoma cells were seeded in 24-well plates 24 h before treatment. PD-L1 silencing was achieved by incubating cells with two sequential doses of PD-L1-siRNA–loaded BMV-VLPs (PDL1-VLPs) at a final siRNA concentration of 50 nM, administered 24 h apart. After the final treatment, cells were washed with PBS and supplied with fresh medium.

OT-I CD8^+^ T cells were isolated from transgenic mouse splenocytes using magnetic negative selection. Purified T cells were maintained overnight in RPMI supplemented with 10 % FBS and cytokines (IL-2 at 100 U/mL; IL-7 and IL-15 at 5 ng/mL each) to support viability and cytotoxic function.

For cytotoxicity assays, PDL1-VLP–treated and untreated tumor cells were co-cultured with cytokine-activated OT-I T cells. B16-OVA co-cultures were performed at a 1:1 effector-to-target ratio, whereas CMT64-OVA assays used a reduced effector pressure (1:10, T cell:tumor ratio) to enable detection of PD-L1–dependent effects. Co-cultures were incubated for 18–24 h.

Following co-culture, non-adherent cells were removed, and tumor cell viability was quantified using the CellTiter-Glo Luminescent Cell Viability Assay (Promega, #G7570) according to the manufacturer's instructions. Luminescence, proportional to intracellular ATP content, was measured using a microplate reader. Viability values were normalized to untreated tumor controls. All experimental conditions were performed in triplicate.

### 4.10 Mixed leukocyte reaction (MLR)

Monocytes were first isolated from human PBMCs as previously described [76]. Isolated monocytes were differentiated into immature dendritic cells (moDCs) by culturing them for 7 days in DMEM low glucose supplemented with 10 % FBS, 500 U/mL recombinant human IL-4 (PeproTech, #200–04), and 250 U/mL recombinant human GM-CSF (Abcam, ab88382), with cytokine-supplemented medium refreshed every 2–3 days.

PBMCs from an unrelated donor were freshly isolated and labeled with CFSE according to the manufacturer's protocol. Labeled PBMCs were co-cultured with monocyte-derived dendritic cells at a 10:1 ratio for 5 days in complete medium containing 1 μg/mL of either Atezolizumab or isolated IgGA Fc-fusion peptide. For experimental conditions, moDCs were pretreated with two sequential doses of human PDL1-siRNA–loaded BMV-VLPs prior to initiating co-culture.

At the end of the culture period, cells from the supernatant were collected, and analyzed by flow cytometry. CFSE dilution, in CD3^+^CD8^+^ T cells, was used to quantify allogeneic T-cell proliferation.

### 4.11 Statistical analysis

All quantitative data are presented as mean ± SD from at least three independent experiments unless otherwise stated. Statistical analyses were performed using OriginPro software (OriginLab Corporation, Northampton, MA, USA). Group comparisons were analyzed using one-way ANOVA followed by Tukey's post hoc test for multiple comparisons. A p-value less than 0.05 was considered statistically significant. Statistical significance is indicated in the figures as follows: *p < 0.05 (∗), p < 0.01 (∗∗), p < 0.001 (∗∗∗), and p < 0.0001 (∗∗∗∗).*

## CRediT authorship contribution statement

**Khalil Elbadri:** Writing – review & editing, Writing – original draft, Visualization, Validation, Methodology, Investigation, Formal analysis, Data curation, Conceptualization. **Manlio Fuscielo:** Writing – review & editing, Visualization, Validation, Supervision, Methodology, Investigation, Formal analysis, Data curation. **Firas Hamdan:** Writing – review & editing, Visualization, Validation, Supervision, Methodology, Investigation, Formal analysis, Data curation. **Ruoyu Cheng:** Writing – review & editing, Visualization, Validation, Methodology, Investigation. **Sara Feola:** Writing – review & editing, Visualization, Validation, Methodology, Investigation. **Honey Bokharaie:** Writing – review & editing, Methodology, Investigation. **Carmine D'Amico:** Writing – review & editing, Methodology, Investigation. **Giuseppina Molinaro:** Writing – review & editing, Methodology, Investigation. **Alexandra Correia:** Writing – review & editing, Methodology, Investigation. **Shiqi Wang:** Writing – review & editing, Visualization, Validation, Supervision, Resources, Project administration, Funding acquisition. **Michael Jeltsch:** Writing – review & editing, Visualization, Validation, Supervision, Resources, Project administration, Funding acquisition. **Vincenzo Cerullo:** Writing – review & editing, Visualization, Validation, Supervision, Resources, Project administration, Funding acquisition. **Hélder A. Santos:** Writing – review & editing, Visualization, Validation, Supervision, Resources, Project administration, Funding acquisition, Conceptualization.

## Declaration of competing interest

The authors declare that they have no known competing financial interests or personal relationships that could have appeared to influence the work reported in this paper.

## Data Availability

Data will be made available on request.
