# Supplementary material for: Design and in vitro validation of Brome mosaic –virus-like particles for gene delivery and immunomodulation of melanoma
Source: Mater Today Bio. 2025 Dec 18;36:102693. doi: 10.1016/j.mtbio.2025.102693 (PMC12813231; doi:10.1016/j.mtbio.2025.102693)
Supplement: Multimedia component 1 [file mmc1.docx]

[Design and in vitro validation of Brome mosaic virus-like particles for gene delivery and immunomodulation of melanoma](https://thessa.helsinki.fi/phd-project/publications/13704)

Khalil Elbadri¹^,*^, Manlio Fuscielo², Firas Hamdan², Ruoyu Cheng^1,5^, Sara Feola³, Honey Bokharaie¹, Carmine D’Amico⁴, Giuseppina Molinaro¹, Alexandra Correia¹, Shiqi Wang^1,5^, Michael Jeltsch^2,6,7,8^, Vincenzo Cerullo², and Hélder A. Santos^9,*^

Affiliations:

¹ Drug Research Program, Division of Pharmaceutical Chemistry and Technology, Faculty of Pharmacy, University of Helsinki, Helsinki FI-00014, Finland
² Drug Research Program, Division of Pharmaceutical Biosciences, Faculty of Pharmacy, University of Helsinki, Viikinkaari 5E, Helsinki FI-00014, Finland
³ Orion Corporation, Turku, Finland
⁴ Department of Bioproducts and Biosystems, Aalto University, 00076 Aalto, Finland

^5^ Institute of Biotechnology, Helsinki Institute of Life Science, University of Helsinki, Helsinki, Finland
^6^ Individualized Drug Therapy Research Program, Faculty of Medicine, University of Helsinki, Helsinki FI-00014, Finland
^7^ Wihuri Research Institute, Helsinki FI-00014, Finland
^8^ Helsinki One Health, University of Helsinki, Helsinki FI-00014, Finland

^9^Department of Biomaterials and Biomedical Technology, The Personalized Medicine Research Institute (PRECISION), University Medical Center Groningen, University of Groningen, Ant. Deusinglaan 1, 9713 AV Groningen, The Netherlands

Corresponding authors: [khalil.elbadry@helsinki.fi](mailto:khalil.elbadry@helsinki.fi); h.a.santos@umcg.nl

**Keywords:** siRNA delivery; Melanoma immunotherapy ; PD-L1 silencing; Plant virus nanocarriers; Protein cages;

## Results and discussion


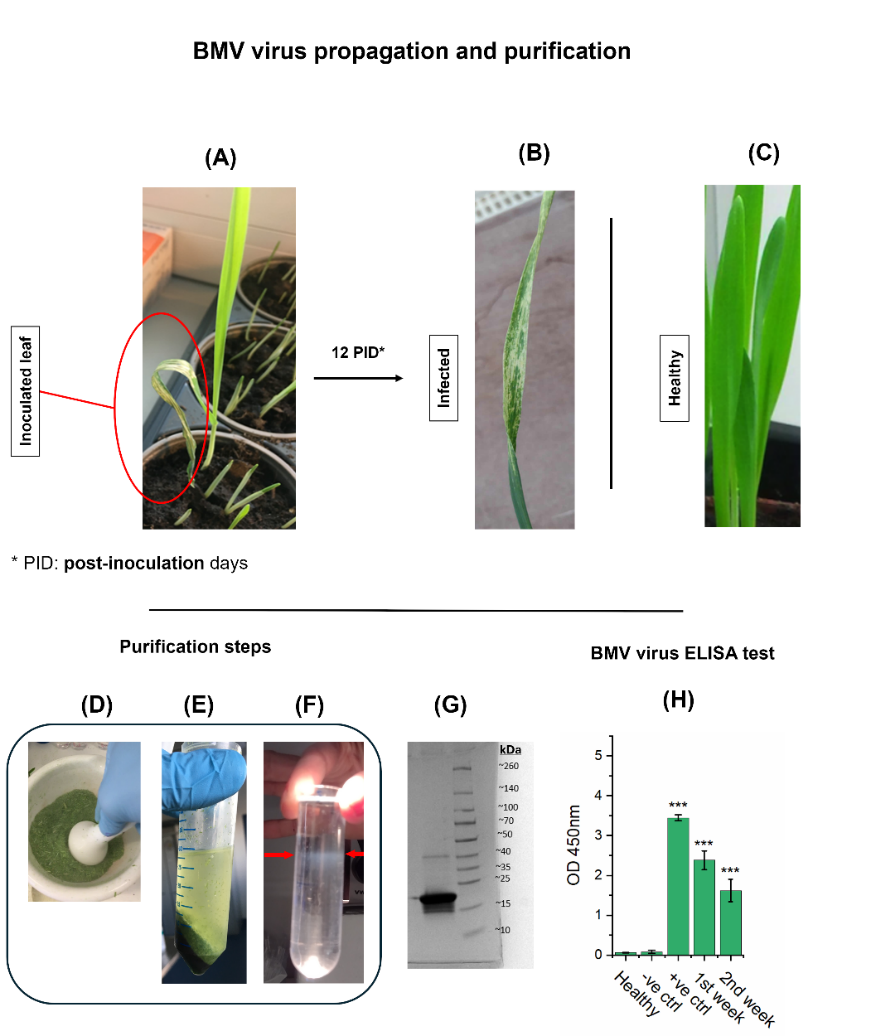


**Figure S1:** Propagation and purification of wtBMV. Hordeum vulgare (barley) plants were mechanically inoculated with homogenates from infected leaves (A). At 12 days post-inoculation (dpi), symptomatic leaves exhibited characteristic chlorotic and mosaic patterns (B), in contrast to healthy leaves (C). (D–F) Virus particles were extracted and purified from symptomatic leaves through differential centrifugation and ultracentrifugation using sucrose cushions and gradients, whereas the final virus band is visible and pointed at by the red arrows (F). (G) SDS-PAGE analysis revealed a clear band (~20 kDa) corresponding to the BMV capsid protein. (H) BMV-specific ELISA confirmed significant viral protein presence in extracts collected at 1- and 2-weeks post-inoculation, whereas +ve control was composed of readily infected leaves (Agdia). Error bars indicate standard deviation and statistical significance is indicated by (***), p < 0.001 compared to healthy control.


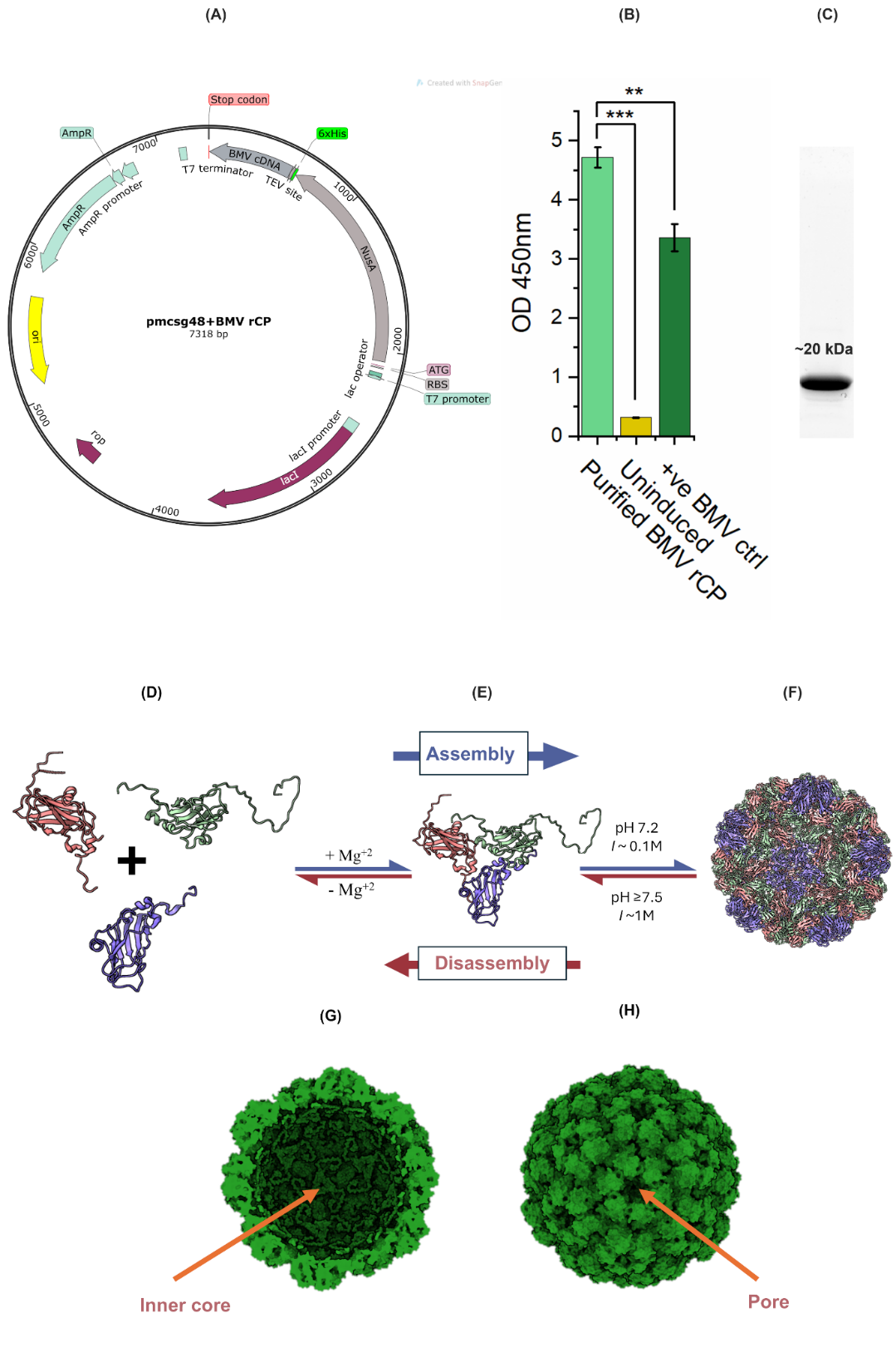


**Figure S2:** Production and structural modeling of recombinant BMV-VLPs. (A) Schematic of the expression construct (pMCSG48) used for E. coli-based production of rCP. (B) ELISA result showing a strong immunoreactivity of recombinant BMV VLPs and wtBMV (as +ve control) against BMV-specific antibodies, with negligible signal from uninduced bacterial lysates. (C) SDS-PAGE analysis of purified recombinant BMV rCP showing a prominent band at ~20 kDa, consistent with the expected size of native coat protein. (D-F) Diagram summarizing the pH, ionic strength and Mg²⁺-dependent reversible assembly/disassembly behavior of BMV capsid proteins: (D) Structural representation of the three quasi-equivalent conformations, A, B, and C (purple, red and green, respectively), adopted by BMV capsid protein monomers during T=3 icosahedral assembly. (E) Schematic illustrating the reversible assembly and disassembly of BMV capsid protein monomers, driven by changes in pH, ionic strength, and Mg²⁺ availability. (F) fully assembled BMV capsid exhibiting T=3 icosahedral symmetry, formed by the organization of 180 monomers in A, B, and C conformations. The assembly is stabilized under near-neutral pH and moderate ionic strength, while disassembly is triggered under alkaline and high-salt conditions. (G,H) Structural models of the BMV-VLP generated based on available crystallographic data, highlighting the icosahedral lattice formed by rCP monomers, the internal cavities and surface pores ideal for nucleic acid packaging and surface functionalization. All 3D structural representations (D–H) were generated using UCSF ChimeraX software.

**SDS-PAGE images**

*
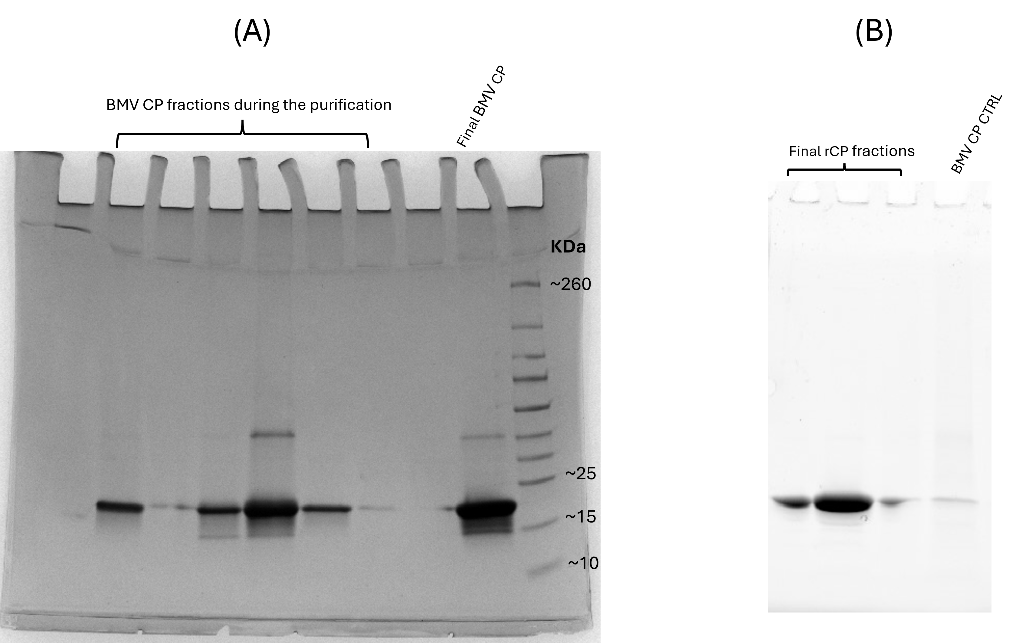
*

**Figure S3:** SDS-PAGE analysis of BMV capsid protein (CP). (A) Analysis of wtBMV factions, collected during the purification process. A distinct ~20 kDa band corresponding to the CP is visible in multiple fractions, with the final purified virions loaded in the last lane before the molecular weight ladder. (B) SDS-PAGE of recombinant BMV CP (rCP) purified from E. coli following TEV protease digestion of the NusA-CP fusion protein, showing a clear ~20 kDa band for the mature CP. The final lane on the right contains wtBMV CP as a reference control**.**

**Cell uptake**

**
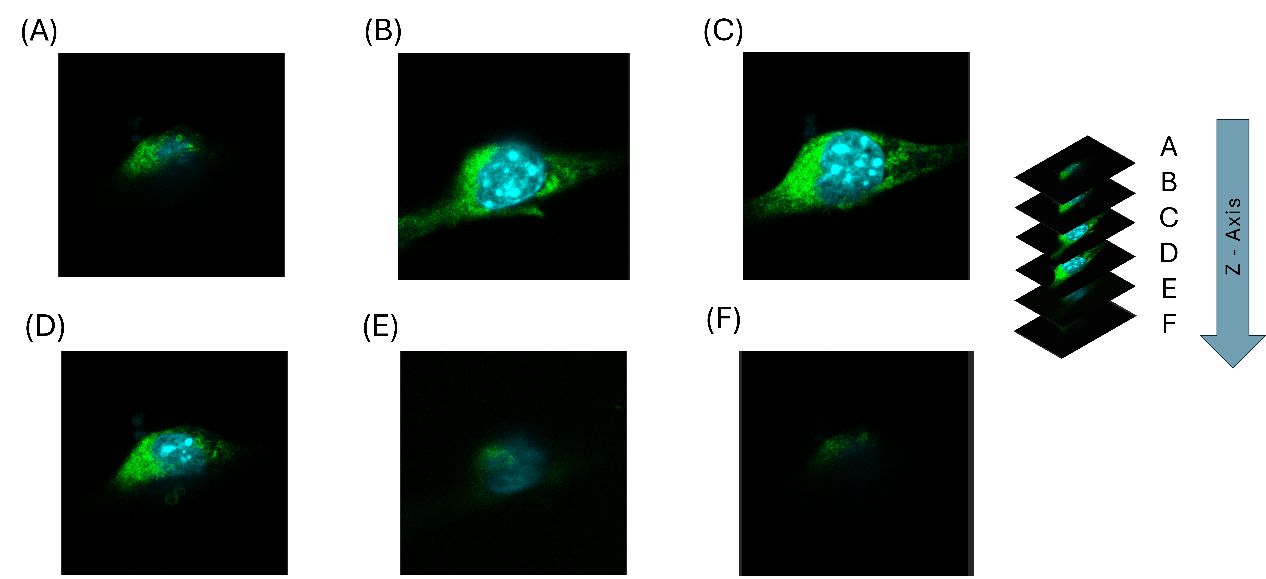
**

**Figure S4:** Confocal Z-stack images showing the intracellular localization of fluorescently labeled BMV particles (green) in B16F10 cells. The nucleus is stained with DAPI (blue). Serial optical sections were acquired along the Z-axis from the top (A, top-left) to the bottom (F, bottom-right) of the cell. The fluorescence signal corresponding to internalized BMV particles is visible throughout the cytoplasm, confirming cellular uptake and intracellular distribution. The schematic on the right illustrates the imaging depth along the Z-axis.

**Fluorescence microscopy images**

**
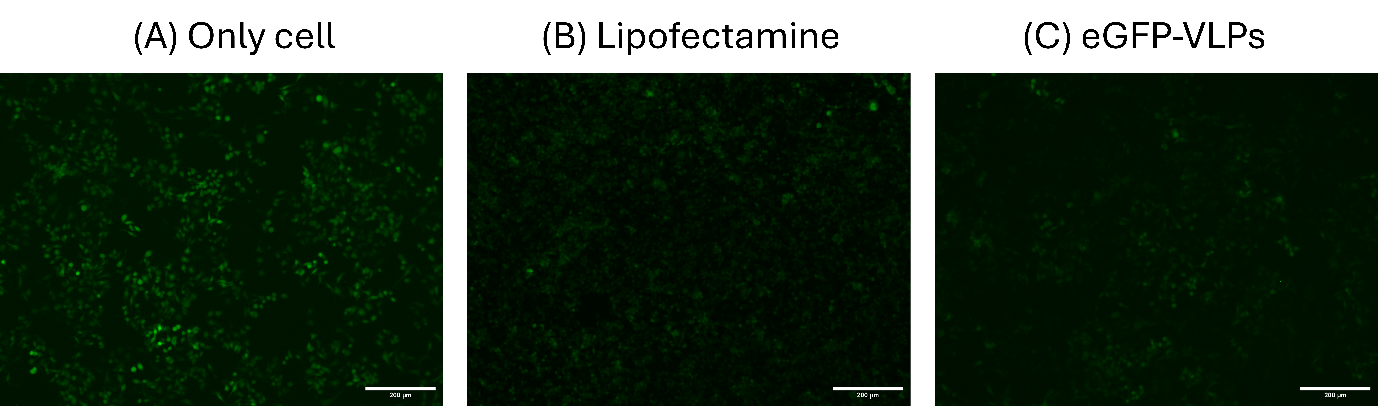
**

**Figure S 5:** Fluorescence microscopy images showing eGFP expression in RAW264.7 cells. (A) Untreated control cells exhibiting strong native eGFP fluorescence. (B) Cells treated with eGFP-siRNA delivered using Lipofectamine, showing reduced fluorescence intensity. (C) Cells treated with eGFP-siRNA encapsulated in BMV-VLPs, also showing reduced fluorescence intensity consistent with eGFP silencing. Scale bars: 200 µm.

## Materials and methods

**GFP sequence:**

ATGGTGAGCAAGGGCGAGGAGCTGTTCACCGGGGTGGTGCCCATCCTGGTCGAGCTGGACGGCGACGTAAACGGCCACAAGTTCAGCGTGTCCGGCGAGGGCGAGGGCGATGCCACCTACGGCAAGCTGACCCTGAAGTTCATCTGCACCACCGGCAAGCTGCCCGTGCCCTGGCCCACCCTCGTGACCACCCTGACCTACGGCGTGCAGTGCTTCAGCCGCTACCCCGACCACATGAAGCAGCACGACTTCTTCAAGTCCGCCATGCCCGAAGGCTACGTCCAGGAGCGCACCATCTTCTTCAAGGACGACGGCAACTACAAGACCCGCGCCGAGGTGAAGTTCGAGGGCGACACCCTGGTGAACCGCATCGAGCTGAAGGGCATCGACTTCAAGGAGGACGGCAACATCCTGGGGCACAAGCTGGAGTACAACTACAACAGCCACAACGTCTATATCATGGCCGACAAGCAGAAGAACGGCATCAAGGTGAACTTCAAGATCCGCCACAACATCGAGGACGGCAGCGTGCAGCTCGCCGACCACTACCAGCAGAACACCCCCATCGGCGACGGCCCCGTGCTGCTGCCCGACAACCACTACCTGAGCACCCAGTCCGCCCTGAGCAAAGACCCCAACGAGAAGCGCGATCACATGGTCCTGCTGGAGTTCGTGACCGCCGCCGGGATCACTCTCGGCATGGACGAGCTGTACAAGTAG

**eGFP siRNA:**

- Sense 5’ CAA-GCU-GAC-CCU-GAA-GUU-C55 3’
- Anti-sense 5’ GAA-CUU-CAG-GGU-CAG-CUU-G55 3’

**PD-L1 cDNA target sequence – PD-L1**

AACCCTCTGATCGTCGATTGGCAGCTTGTGGTCTGTGAAAGAAAGGGCCCATGGGACATGAGTCCAAAGACTCAAGATGGAACCTGAGGGAGAGAACCAAGAAAGTGTTGGGAGAGGAGCCTGGAACAACGGACATTTTTTCCAGGGAGACACTGCTAAGCAAGTTGCCCATCAGTCGTCTTGGGAAATGGATTGAGGGTTCCTGGCTTAGCAGCTGGTCCTTGCACAGTGACCTTTTCCTCTGCTCAGTGCCGGGATGAGAGATGGAGTCATGAGTGTTGAAGAATAAGTGCCTTCTATTTATTTTGAGTCTGTGTGTTCTCACTTTGGGCATGTAATTATGACTGGTGAATTCTGACGACATGATAGATCTTAAGATGTAGTCACCAAACTCAACTGCTGC

**MISSION® esiRNA** (Sigma-Aldrich, Germany) are endoribonuclease-prepared small interfering RNAs (siRNAs). They consist of a heterogeneous mixture of siRNA fragments, all targeting the same PD-L1 mRNA sequence. According to the manufacturer, this multi-targeting approach enhances both specificity and efficacy of gene silencing.
